# Unraveling Microbial Volatile Elicitors Using a Transparent Methodology for Induction of Systemic Resistance and Regulation of Antioxidant Genes at Expression Levels in Chili against Bacterial Wilt Disease

**DOI:** 10.3390/antiox11020404

**Published:** 2022-02-16

**Authors:** Abhijeet Shankar Kashyap, Nazia Manzar, Suresh M. Nebapure, Mahendra Vikram Singh Rajawat, Man Mohan Deo, Jyoti Prakash Singh, Amit Kumar Kesharwani, Ravinder Pal Singh, S. C. Dubey, Dinesh Singh

**Affiliations:** 1 Division of Plant Pathology, ICAR-Indian Agricultural Research Institute, New Delhi 110012, India; amitmicro1@gmail.com (A.K.K.); ravinder.20033@gmail.com (R.P.S.); 2 Plant Pathology Laboratory, ICAR-National Bureau of Agriculturally Important Microorganisms, Maunath Bhanjan 275103, India; naziamanzar786@gmail.com (N.M.); rajawat.mvs@gmail.com (M.V.S.R.); jyotipsingh58@outlook.com (J.P.S.); 3 Division of Entomology, ICAR-IARI, New Delhi 110012, India; nebapure.mahadev@icar.gov.in; 4 Farm Machinery and Power, ICAR-Indian Institute of Pulses Research, Kanpur 208024, India; mm.deo@icar.org.in; 5 Division of Biochemistry, ICAR-Indian Agricultural Research Institute, New Delhi 110012, India; 6 Division of Plant Quarantine, ICAR-NBPGR, New Delhi 110012, India; scdube2002@yahoo.co.in; 7 Krishi Bhawan, Indian Council of Agricultural Research, New Delhi 110001, India

**Keywords:** *B. subtilis* KA9, volatile compounds, GC–MS spectroscopy, systemic resistance, *P. fluorescens* PDS1, real-time PCR, transmission electron microscopy, *Ralstonia solanacearum*

## Abstract

Microbial volatiles benefit the agricultural ecological system by promoting plant growth and systemic resistance against diseases without harming the environment. To explore the plant growth-promoting efficiency of VOCs produced by *Pseudomonas fluorescens* PDS1 and *Bacillus subtilis* KA9 in terms of chili plant growth and its biocontrol efficiency against *Ralstonia solanacearum*, experiments were conducted both in vitro and in vivo. A closure assembly was designed using a half-inverted plastic bottle to demonstrate plant–microbial interactions via volatile compounds. The most common volatile organic compounds were identified and reported; they promoted plant development and induced systemic resistance (ISR) against wilt pathogen *R. solanacearum*. The PDS1 and KA9 VOCs significantly increased defensive enzyme activity and overexpressed the antioxidant genes PAL, POD, SOD, WRKYa, PAL1, DEF-1, CAT-2, WRKY40, HSFC1, LOX2, and NPR1 related to plant defense. The overall gene expression was greater in root tissue as compared to leaf tissue in chili plant. Our findings shed light on the relationship among rhizobacteria, pathogen, and host plants, resulting in plant growth promotion, disease suppression, systemic resistance-inducing potential, and antioxidant response with related gene expression in the leaf and root tissue of chili.

## 1. Introduction

Bacterial wilt is a catastrophic soil-borne disease that affects nearly 450 crop species, primarily those belonging to the Solanaceae family [1,2]. Bacterial wilt is found all over the world in tropical and subtropical climates [3]. In the management of soil-borne plant diseases, the biological control approach is an environmentally benign, cost-effective, and simple-to-implement strategy. Plant growth-promoting bacteria are considered the greatest biocontrol agents, with the ability to inhibit the population of pathogenic microbes and induce systemic resistance in plants against disease [4]. Antimicrobial compound production, nitrogen fixation, phytohormone production, and mineral solubilization are some of the mechanisms involved in plant growth promotion [5,6,7], including volatile organic compounds (VOCs) [8,9,10,11]. Plant growth-promoting rhizobacteria (PGPR) produce volatile organic compounds (VOCs), which are gaseous metabolic chemicals exhaled from bacterial cells that are active even at a low concentration under normal conditions [12]. The VOCs produced by PGPRs are beneficial in suppressing plant infections, increasing plant development, and establishing systemic resistance [10,13,14,15,16]. VOCs offer an advantage over other biocontrol and growth-regulating mechanisms in that they do not necessitate physical contact with pathogens or plant components, whereas most other methods for suppressing phytopathogens and boosting plant development must [4,5]. Bacterial VOCs can have direct antibacterial action. Many plant pathogen biocontrol agents, such as *Pseudomonas* and *Bacillus*, have been identified to release VOCs exhibiting antibacterial action [5,8,9,10,11,12,17]. For example, VOCs generated by *Bacillus* spp., such as benzaldehyde, 1,2-benzisothiazol-3(2*H*)-one, and 1,3-butadiene, were found to have a substantial inhibitory effect against *R. solanacearum*, which causes bacterial wilt disease [10]. It was reported in many studies that genera *Bacillus*, *Pseudomonas*, *Serratia*, and *Arthrobacter* are linked to plant growth promotion and the induction of systemic resistance [13,14,18,19]. The plant growth-promoting VOCs 2,3-butanediol and acetoin were found to be produced by *Bacillus subtilis* GB03 and *B. amyloliquefaciens* IN937 [10]. In addition to boosting growth, VOCs induce systemic resistance in plants, resulting in tolerance to both biotic and abiotic factors. A *Bacillus* VOC 2,3-butanediol was demonstrated to greatly improve resistance in *Arabidopsis* against *Erwinia carotovora* subsp. *carotovora* [20]. 3-Pentanol and 2-butanone were also reported to be effective against the pathogen *Pseudomonas syringae* pv. *lachrymans*, which causes cucumber bacterial angular leaf spot [21]. The effects of bacterial VOCs on plant development and systemic resistance have been studied in the literature. Bacterial VOCs were shown in some experiments to interact with plant hormones by interfering with morphogenetic processes, causing the plant to develop more quickly [10,13,22,23]. After being exposed to *Bacillus subtilis* GBO3 VOCs, a transcriptional study of *Arabidopsis* revealed that the VOCs can regulate auxin and result in the commencement of growth promotion [22]. Furthermore, VOCs influenced the transcriptional expression of genes related to the ethylene response factor and ethylene production, such as WRKY, implying that VOCs can promote plant disease resistance in poplar [24]. *Bacillus* species are the most effective PGPR bacteria because they can produce spores that survive in harsh environments [25]. The major goal of this study was to investigate the impact of VOCs produced by *Bacillus strain* KA9 and *Pseudomonas* strain PDS-1 on plant growth promotion, induction of systemic resistance, and gene transcriptional expression in chili.

## 2. Materials and Methods

### 2.1. Plant Growth-Promoting Rhizobacteria Strains

Rhizobacteria and *R. solanacearum* cultures used in this study were taken from the Plant Bacteriology Lab, Division of Plant Pathology, Indian Council of Agricultural Research—Indian Agricultural Research Institute, New Delhi, with accession numbers MT491101 and MN368159. The rhizobacteria cultures utilized in this investigation were collected from diverse agroclimatic zones, characterized in 2019, and kept in the lab in glycerol stock, slants, and active plates. These studies were run since 2019 and repeated three times to ensure a valid and stable hypothesis. These cultures were grown in TSA medium (tryptone 17.0 g/L, soya peptone 3.0 g/L, sodium chloride 5.0 g/L, dextrose 2.5 g/L, dipotassium hydrogen phosphate 2.5 g/L, and agar 15.0 g/L), KB medium (proteose peptone 20.0 g/L, dipotassium hydrogen phosphate 1.50 g/L, magnesium sulfate heptahydrate 1.50 g/L, and agar 20.0 g/L), CPG medium (casamino acid 1 g/L, peptone 10 g/L, glucose 5 g/L, and agar 17 g/L, supplemented with 2,3,5-triphenyl tetrazolium chloride at 0.005%) [26]. A single isolated colony of bacterium (irregular, gray-white, round, opaque, thick ridges, smooth, moist, medium-sized colony) was used for growth on a YGCA (yeast glucose calcium carbonate agar) slant. All cultures were maintained on the YGCA slant and stored at 4 °C for further use.

### 2.2. Detection of Volatile Inhibitory Compounds Produced by Rhizobacteria against R. solanacearum under In Vitro Conditions

**Figure 1:**
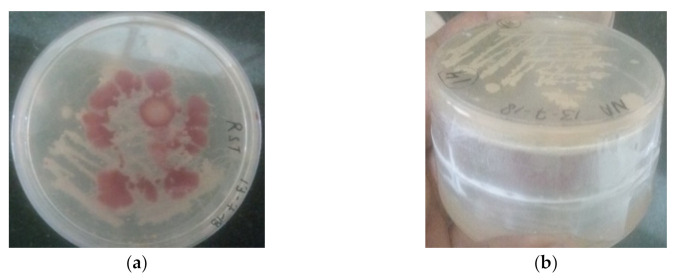
(**a**) Double-plate assay for the detection of volatile inhibitory compounds produced by rhizobacteria against *R. solanacearum* under in vitro conditions; (**b**) lateral view of sealed plates carrying antagonists in the lower plate and pathogens in the upper plate.

A dual-plate assay was first used to determine the potential of rhizobacterial isolates PDS1 and KA9 in producing volatile inhibitory compounds. A colony of each rhizobacterium was smeared on an NA plate, and the same process was repeated for *R. solanacearum* on a separate CPG plate. The agar plate covers were then removed, and the *R. solanacearum*-inoculated plate was flipped over the rhizobacteria-inoculated plate (Figure 1), whereby the sealed plates featured antagonists in the lower plate and pathogens in the upper plate. Parafilm was used to seal the Petri plates together before they were placed in an incubator at 25 °C for 7 days. Control plates were established by inverting *R. solanacearum*-inoculated plates over a plain agar plate (designated as D1 in this experiment). The growth of *R. solanacearum* in control was compared against its growth in the presence of the rhizobacteria (designated as D2).

The inhibition (%) by the rhizobacteria was calculated using *D*1 and *D*2 (Equation (1)).

(1) $$Growth\;inhibition=\frac{D1-D2}{D1}\times 100,$$

where *D*1 is the diameter growth of *R. solanacearum* only (control), and *D*2 is the diameter growth of *R. solanacearum* co-inoculated with rhizobacteria.

### 2.3. Study on Induced Systemic Resistance (ISR) of Rhizobacteria against R. solanacearum in Chili Plants

#### 2.3.1. Rhizobacterial Cultures and Chili Seedlings

**Figure 2:**
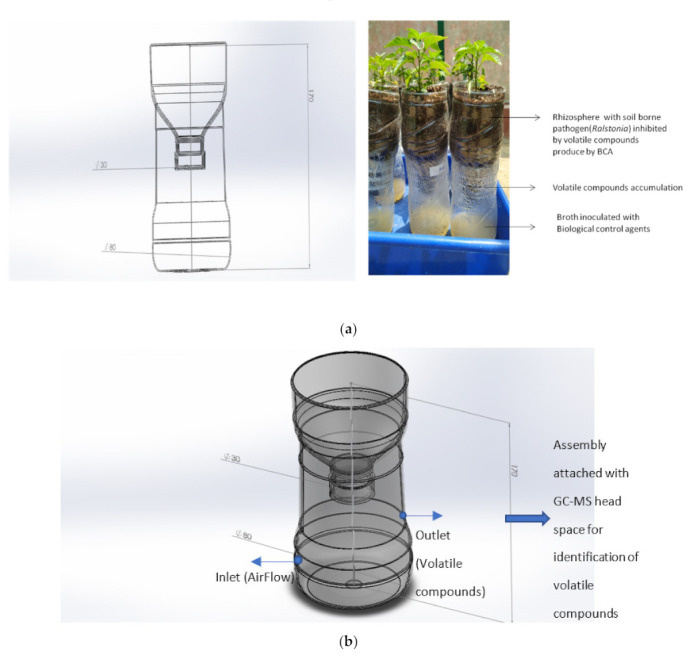
(**a**,**b**) A low-cost closed assembly resembling a transparent pot system using a half-inverted plastic bottle to demonstrate plant–microbial interactions via volatile compounds.

Rhizobacterial isolates *Pseudomonas fluorescens* (PDS1) and *Bacillus subtillis* (KA9) were cultured on a sterile LB agar plate at 28 ± 1 °C for 48 h. They were diluted with sterile distilled water to 10^8^ CFU/mL. The pathogen was grown on CPG agar medium at 28 ± 1 °C for 48 h, and the inoculum load was maintained at 10^8^ CFU/mL. Chili cv. Pusa Jwala seeds were planted in seed starter trays with 96 wells containing a 2:1:1 mixture of peat mass, vermiculite, and sand in a Phytotron under controlled conditions at 28 ± 2 °C, watered daily. As illustrated in Figure 2, the lower part of the closer assembly contained broth for the optimum growth of biocontrol agents producing the volatile compounds transmitted to the upper part of bottle, which interfered with the pathogen *R. solanacearum* present in the rhizospheric zone of the upper part of the assembly where the chili was transplanted. The plants were simultaneously treated with rhizobacteria and *R. solanacearum* UTT-25 5 days after transplantation. After 48 h, cultures of wilt pathogen and biocontrol agents were scraped from the Petri plates and mixed in sterile distilled water to maintain the bacterial population at 0.1 OD (600 nm) according to a spectrophotometer. Then, a 2 mL suspension of *R. solanacearum* was inoculated in the rhizospheric zone after 5 days of transplanting. Subsequently, 5 mL of antagonists were inoculated in the lower chamber of the assembly. The plants treated with pathogen *R. solanacearum* only and uninoculated plants were also maintained as positive and negative controls, respectively. The observations were recorded at 7 day intervals for 40 days after transplanting. Three replicates were maintained for the experiment.

The seven treatments were as follows: T-1 (control), T-2 (*B. subtilis* KA9), T-3 (*P. fluorescens* PDS1), T-4 (*B. subtilis* KA9 + *R. solanacearum* UTT-25), T-5 (*P. fluorescens* PDS1 + *R. solanacearum* UTT-25), T6 (*P. fluorescens* PDS1 + *B. subtilis* KA9 + *R. solanacearum* UTT-25), and T-7 (*R. solanacearum* UTT-25). The surviving plants were assessed for wilt intensity and fresh and dry weight at the time of harvesting. The degree of wilt was studied at 7 day intervals for 60 days. The average percentage of wilt in each treatment was calculated by following the scale of disease severity, where 1 indicates no symptoms, 2 indicates one wilted leaf, 3 indicates two to three wilted leaves, 4 indicates four or more wilted leaves, and 5 indicates that the entire plant has wilted (dead plant). Wilt incidence was estimated using the 30 days after inoculation according to the following formula [26]:

Wilt intensity (%) (I) = [∑ (n_i_ × v_i_) ÷ (V × N)] 100,

where n_i_ is the number of plants with a specific disease rating, v_i_ is the disease rating (1, 2, 3, 4, or 5), V is the highest disease rating, and N is the number of plants observed. Biocontrol efficacy was calculated as described in [27]. Growth-promoting efficacy was calculated as a function of the dry weight of the root and shoot of the plant, as described in [28].

#### 2.3.2. Preparation for Enzyme Extraction

Approximately 0.5 g of leaf tissue was collected from chili cv. Pusa Jwala for enzyme extraction under each treatment at timepoints of 0, 24, 48, 72, and 96 h. Leaf tissue and root tissue were homogenized in 0.1 M sodium phosphate buffer (20 mL), β-mercaptoethanol (0.2 mL), and 1.0 g of insoluble polyvinyl pyrrolidone (PVPP), centrifuged at 12,000 rpm for 30 min at 4 °C. In the supernatant, the concentration of ammonium sulfate was measured. Extraction buffer (3 mL) was used to recover the pellets from the supernatant after 1 h of centrifugation at 12,000 rpm for 30 min at 40 °C, and the pellets were utilized for assays to evaluate defense enzymes such as SOD, POD, PPO, and PAL.

#### 2.3.3. Estimation of PPO Activity

The polyphenol oxidase activity was determined according to the method described in [29]. The reaction mixture contained potassium phosphate buffer (2 mL), pyrogallol (1 mL), and 0.1 mL of the enzyme extract, kept at room temperature for 3 min for incubation. The reaction was terminated by adding 0.5 mL of sulfuric acid, and the OD at 480 nm was measured immediately using a UV/Vis spectrophotometer (Hitachi, U-2900) against the control of potassium phosphate buffer (2 mL) and pyrogallol (1 mL). Sulfuric acid (0.5 mL) was added and incubated for 3 min at room temperature. The enzyme extract (0.1 mL) was added after 3 min of incubation, before immediately recording the OD value. The reaction mixture without the enzyme served as the blank.

One unit of enzyme activity was described by a change in absorbance of 0.1 per minute. The specific activity was calculated using the following formula:

(A × 3/0.1)/3 = specific activity/min/mg protein,

where A is the difference in absorbance of the sample and the control (OD S − OD C).

#### 2.3.4. Estimation of POD Activity

Peroxidase activity was determined using the method described in [29]. Briefly, 1 mL of hydrogen peroxide (H_2_O_2_), 2 mL of potassium phosphate buffer, and 1 mL of pyrogallol were combined. The enzyme extract (0.1 mL) was added to the mixture and left to rest for 3 min at room temperature. Then, 0.5 mL of sulfuric acid was added to terminate the reaction. The OD at 420 nm was measured for 5 min, and one unit of enzyme activity was defined as a change in absorbance of 0.1 per minute. The specific activity was calculated using the following formula:

(A × 3/0.1)/3 = specific activity/min/mg protein,

where A is the difference in absorbance of the sample and the control (OD S − OD C).

#### 2.3.5. Estimation of SOD Activity

Superoxide dismutase activity was determined using the NBT method described by Beauchamp and Fridovich [30]. First, 0.1 M phosphate buffer (pH 7.8), 13 mM methionine, 0.1 mM EDTA, 75 M NBT, 0.1 mL of enzyme extract, and 2 M riboflavin were combined in a 3.0 mL reaction mixture. The test tubes containing the reaction mixture were placed in an incubator for 10 min and shaken in the light. The reaction was halted by turning off the light and covering the tubes with a black cloth. The OD at 560 nm was used to quantify SOD activity. A 50% reduction in NBT was denoted as one unit of enzyme activity according to the OD value of the reaction solution. The specific activity of SOD was calculated using the following formula:

(100 − (OD S/OD LC) 100)/50 = *x*,

where *x* is the specific activity per mg protein in the enzyme extract. One unit of enzyme activity was defined as a 50% reduction in the blue color formed by NBT/30 min/mg protein. OD S = OD T − OD C, where OD T is the absorbance of the sample, OD C is the absorbance of the dark control, and OD LS is the absorbance of the light control.

#### 2.3.6. Estimation of PAL Activity

Chili leaves and roots (0.2 g each) of cv. Pusa Jwala were homogenized in 2 mL of 25 mM borate buffer (100 mM borate and 150 mM NaCl, pH 8.8) containing 2 μL of β-mercaptoethanol and a trace amount of polyvinyl polypyrrolidone (PVP). The homogenate was filtered through a 0.45 μm nylon membrane filter and centrifuged at 12,000× *g* for 10 min, and the supernatant was used for the enzyme activity assay as described by Sadisivam and Manickam [31]. One unit of the enzyme activity was defined as a one unit increase in absorbance per minute. The OD at 290 nm was measured every 15 min until it became constant. A change in absorbance of 0.1 per hour was denoted as one unit of enzyme activity.

### 2.4. Colonization Behavior of Different Groups of Rhizobacteria and R. solanacearum on Chili Root

After 30 days of inoculation, the levels of *R. solanacearum* and rhizobacteria in the plant system were assessed. Three chili plants were randomly selected from each treatment, and 1 g of the root was crushed in 5 mL of a 0.85% brine solution, before diluting serially. To determine the growth of *R. solanacearum* and rhizobacteria, a 100 µL aliquot was disseminated equally over TTC and LA media. The infected Petri plates were incubated at 28 °C for 48 h. The number of colony-forming units (CFU) per gram of plant fresh weight was counted. The trials were carried out in a glass house in triplicate under identical conditions, and the data were pooled for statistical analysis.

### 2.5. ISR Activity of Rhizobacteria VOCs in a Half-Inverted Plastic Bottle Representing an Earth System Assembly

A low-cost closed assembly resembling an Earth system was established using a half-inverted plastic bottle to demonstrate plant–microbial interactions via volatile compounds. This system collects microbial volatile compounds from the interior of an inverted plastic bottle, featuring a downward-pointing spout covered with a muslin cloth. The closure has an open pore through which volatile compounds may flow from the inverted plastic bottle (Figure 2). The lower part of the assembly contained broth for the optimum growth of biocontrol agents producing the volatile compounds transmitted to the upper part of the assembly, where they interfered with the pathogen *R. solanacearum* present in the rhizospheric zone. The jar assembly was placed at 25 °C in a 12 h light/12 h dark photoperiod for 30 days. The induction of systemic resistance was determined by measuring the difference in regulation of defensive enzymes PAL, PPO, POD, and SOD in the root and shoot tissues of seven different treatments. The seven treatments were as follows: T-1 (uninoculated control treated with sterile water only), T-2 (*B. subtilis* KA9), T-3 (*P. fluorescens* PDS1), T-4 (*B*. *subtilis* KA9 + *R. solanacearum* UTT-25), T-5 (*P. fluorescens* PDS1 + *R. solanacearum* UTT-25), T6 (*P. fluorescens* PDS1 + *B. subtilis* KA9 + *R. solanacearum* UTT-25), and T-7 (*R. solanacearum* UTT-25 only).

### 2.6. GC/MS Analysis of Volatile Compounds Produced by Rhizobacteria Strains

The separation and identification of volatile compounds from the plastic bottle assembly were achieved using a Shimadzu QP 2000 fitted with an Rtx-5ms column (30 m × 0.25 mm) packed with 95% dimethyl poly siloxane for GC–MS analysis. Helium with a flow rate of 1 mL/min was used as the carrier gas. First, 1.0 μL of each sample was injected into the injection port with temperature maintained at 230 °C. The initial temperature of the oven was programmed at 40 °C for 4 min, before it was increased to 220 °C with 5 °C ramping rate and held for 2 min. Finally, the temperature was increased to 270 °C with a ramping rate of 15 °C and held for 1 min. The running time of the sample was 45 min. The ion source was kept at a constant temperature of 200 °C. For GC–MS analysis, electron impact ionization (EII) at 70 eV was utilized, and the data were analyzed using TIC (total ion count) for compound identification and quantification. The spectra of each molecule were compared to a recognized spectral database in the GC–MS library (NIST14), and the data were then processed by measuring the peak regions using Turbo-Mass-OCPTVS-Demo SPL software.

### 2.7. Population Counts of R. solanacearum in Pot Experiments with Chili

The roots of the chili plants cv. Pusa Jwala were collected. Then, 5 mL of 0.85% brine solution was used to crush 1.0 g of root, before diluting serially. To determine rhizobacteria growth, a 100 µL aliquot was evenly disseminated on Luria agar medium. The inoculated Petri plates were incubated at 28 ± 1 °C for 48 h. The colonies were enumerated to compute the number of colony-forming units (CFU) per gram of plant fresh weight.

### 2.8. Expression of Rhizobacteria-Mediated Defense-Related Genes against R. solanacearum in Chili

#### 2.8.1. RNA Isolation

First, 100 mg of fresh root and leaf samples were carefully gathered from the infected spot (about 10 mm) and homogenized in liquid nitrogen. With an RNA isolation kit, total RNA was extracted from the tissues using the silica spin column technique to obtain pure and undamaged RNA (Genei, Bangalore, India) (Supplementary Materials).

#### 2.8.2. Quantification of the Isolated RNA

Diluting the samples with RNase-free water allowed the RNA content to be quantified. The absorbance of the samples was measured at 260 and 280 nm, where 40 g/mL RNA equates to an absorbance reading of 1.0 at 260 nm. The A260/A280 ratio was used to determine the sample purity. The RNA purity is judged satisfactory for values >1.6.

#### 2.8.3. Electrophoretic Separation of RNA

The following reagents /buffers were used: 0.8% agarose, 1 mM EDTA, 40 mM Tris acetate, 0.25% bromophenol blue, 0.25% xylene glycol, and 30% glycerol. The 0.8% agarose was melted in 1× TAE buffer (50× TAE buffer (40 mM Tris acetate, pH 7.5 and 1 mM EDTA) diluted with DEPC-water). Then, 0.5 µg/mL ethidium bromide (EtBr) was added when the mixture cooled to around 60 °C. The gel medium was placed onto horizontal gel trays with combs to generate vertical gel loading pockets for solidification. The polymerized gels were placed into an RNase-free gel tank with 1× TAE buffer. Next, 2 μL of RNA was placed into the wells using 6× loading buffer (0.25% (*w*/*v*) bromophenol blue, 0.25% (*w*/*v*) xylene glycol, and 30% (*v*/*v*) glycerol). The RNA was separated on the gels at 100 V for 1 h. The fluorogram was documented photographically using a gel documentation system (Alpha Imager, Bengaluru, Karnataka, India). 

#### 2.8.4. cDNA Synthesis

An M-MuLV Reverse Transcriptase kit was used to obtain cDNA from whole RNA (Genei, Bangalore, India). The kit’s components were thawed, combined, and briefly centrifuged before being stored on ice. The following reagents were introduced in order to a sterile, nuclease-free tube on ice: 5 g of total RNA, 1 μL of oligo (dT) primer, and 12 μL of nuclease-free water. The reagents were gently mixed, centrifuged at 2500 rpm, and incubated at 65 °C for 5 min. The tube was chilled on ice before being spun down and then returned to the ice. Next, 4 µL of 5× reaction buffer, 1 µL of RNase inhibitor, 2 µL of 10 mM dNTP Mix, and 1 µL of M-MuLV Reverse Transcriptase, made up to a total volume of 20 µL were mixed gently and centrifuged at 2500 rpm for 5 min. The samples were incubated for 60 min at 42 °C for gene-specific primed cDNA synthesis. The process was stopped after 5 min of heating at 70 °C.

#### 2.8.5. RT-PCR Analysis

Primer pairs specific to chili defensive genes were designed and used. Primers for chili ubiquitin E3 gene (NCBI accession number XM_016720450.1) cDNA (sense, 5’–GTCCATCTGCTCTCTGTTG–3′; antisense, 5′–ACCCCAAGCACAATAAGAC–3′) were used as an internal control. All RT-PCR experiments were conducted in three replicates. Quantitative RT-PCR analysis was conducted to profile the expression of selected defensive genes. The expression of chili genes representing the upstream and downstream pathogenesis response system was profiled by qRT-PCR after infection of chili seedlings with *R. solanacearum* and rhizobacteria. The expression levels of Lox2, UBI-3, POD, SOD, WRKYa, PAL1, DEF1, CAT2, HSFC1, WRKY40, PAL, NPRI, and UBI-3 genes were determined at 48 h post inoculation using real-time PCR, by establishing a gradient concentration using 40 nM of primers and 10–100 ng of cDNA. The PCR conditions involved initial activation at 95 °C for 5 min, denaturation at 95 °C for 30 s, and combined annealing/extension at 60 °C for 30 s. The PCR product was melted at 72 °C for 1 min and then ramped from 72 to 98 °C by 1 °C every 5 s.

The following formula was used to compute the relative gene expression in terms of fold change: fold change = 2 − ΔΔCt where, ΔCt = Ct, target – Ct, normalizer, and ΔΔCt = ΔCt, stimulated − ΔCt, control.

### 2.9. Statistical Analysis

All experiments were performed in a completely randomized design with three replicates. Prior to statistical analysis, all bacterial counts were logarithmically converted. One- and two-way analyses of variance (ANOVAs) using SAS (SAS Institute 1998) were applied to evaluate significant differences between treatments at a significance level of 0.05. Tukey’s post hoc multiple comparison test [32] was used to examine differences between experimental treatments. The values of enzyme activities were expressed as the means of three measurements (±SE) from three plants under the same treatment.

## 3. Results

### 3.1. Inhibitory Effect of Volatile Compounds Produced by Rhizobacterial Isolates of Chili against Ralstonia solanacearum

**Figure 3:**
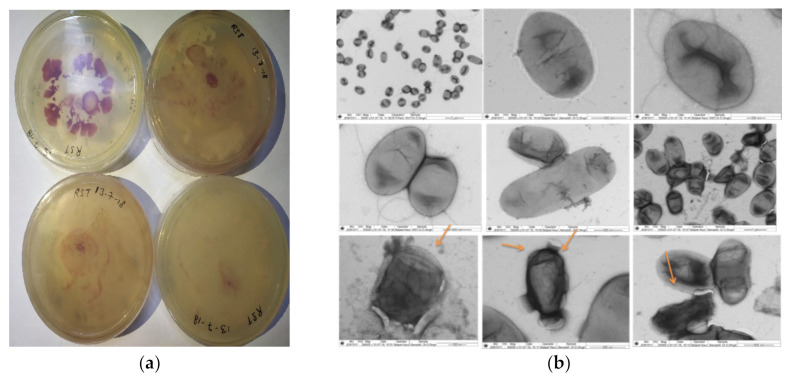
(**a**) Inhibitory effect of volatile compounds produced by PGPRs of chili against *Ralstonia solanacearum* under in vitro conditions. (**b**) TEM images revealing disruption of *R. solanacearum* cells due to volatile compounds produced by rhizobacteria.

We obtained evidence that PGPRs were involved in inhibiting the growth of *R. solonacearum* in our previous study [33]. Nevertheless, to examine how microbial volatile compounds can impact the growth of *R. solonacearum* cells, we examined the pathogen growth under the influence of rhizobacteria producing volatile compounds. It was found after 7 days of observation that the maximum inhibition was achieved by the PDS1 isolate (72.24%), followed by KA9 (68.38%), BDS1 (66.04%), UK2 (64.35%), and UK4 (61.48%) (Table 1). Electron microscopy revealed that most of the *R. solanacearum* cells became irregularly shaped due to disintegration of the cell wall, with most of the cells bursting and dying in the presence of volatile compounds secreted from the PGPR strains (Figure 3).

### 3.2. Analysis of Volatile Compounds Produced by Rhizobacteria under In Vitro Conditions

**Figure 4:**
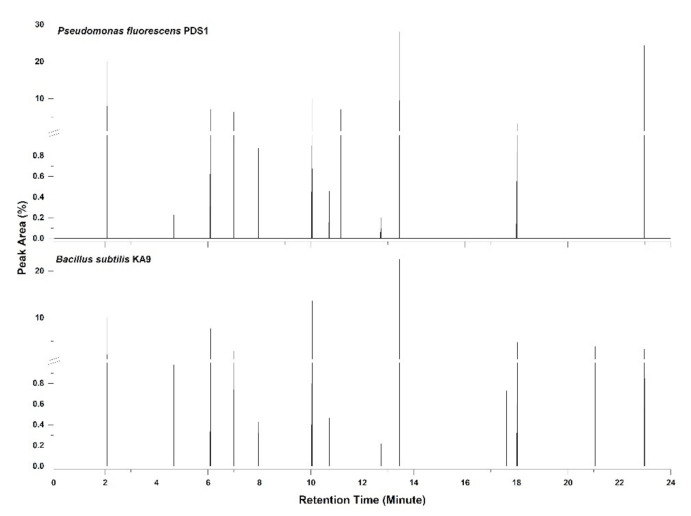
Graph showing gas chromatography–mass spectrometry (GC–MS) profile of major volatile organic compounds (VOCs) produced by rhizobacteria against *R. solanacearum* under in vitro conditions.

A comparative study of the production of volatile compounds by isolates *Pseudomonas fluorescens* PDS1 and *Bacillus subtilis* KA9 was conducted using the GC–MS method. A total of 63 compounds were identified from the GC−MS analysis. The predominant compounds produced by *Bacillus subtilis* strain KA9 in terms of peak area were 2-ethyl-3,6-dimethyl pyrazine (22.59%), 3-hydroxy-2-butanone (acetoin) (13.68%), benzaldehyde (0.32%), cycloheptasiloxane (0.73%), 2-methyl pyrazine (15.95%), diethyl phthalate (7.75%), cyclohexanone (0.22%), 3-isobutyl hexahydropyrrolo (3.96%), 4-hydroxy-5-methyl-2-hexanone (0.43%), 2,4-di-*tert*-butylphenol (0.47%), hexadecane (0.98%), hexahydropyrrolo, (1,2,α) pyrazine-1,4-dione (4.78%), isoamyl alcohol (10.07%), and disulfide dimethyl (2.87%). The predominant compounds produced by *Pseudomonas fluorescens* strain PDS-1 were 2-ethyl-3,6-dimethyl pyrazine (28.07%), 3-hydroxy-2-butanone (acetoin) (10.02%), benzaldehyde (0.43%), 2-methyl pyrazine (24.40%), 1-undecene (7.10%), diethyl phthalate (7.10%), cyclohexanone (0.20%), 3-isobutyl hexahydropyrrolo (0.91%), 4-hydroxy-5-methyl-2-hexanone (0.87%), 2,4-di-*tert*-butylphenol (0.46%), hexadecane (0.23%), hexahydropyrrolo, (1,2,α) pyrazine-1,4-dione (3.23%), isoamyl alcohol (20.09%), and disulfide dimethyl (6.34%). Most compounds were benzene derivatives, with pyrazine-1,4-dione, 3-hydroxy-2-butanone (acetoin), 2-ethyl 3, 6-dimethyl pyrazine, 2-methyl pyrazine, 2-ethyl-3,6-dimethyl pyrazine, and diethylphthalate having peak areas between 2.6% and 28.07% (Figure 4 and Supplementary Table S11). Other volatile molecules were also present but observed in lower concentrations, as indicated by the smaller peak areas (Supplementary Figures S1 and S2).

By analyzing these mass spectra to that in the NIST Mass Spectral Library (probability-based similarity >85%), the chemicals potentially produced by rhizobacteria treatments were identified. The relative content of each compound is shown as a mean (n = 3). The peak area of each compound was calculated as a percentage relative to the total peak area of all volatile organic compounds in a particular treatment. Several minor peaks were not included.

### 3.3. Effect of Volatile Compounds Produced by Rhizobacteria in the Induction of Defensive Enzymes against Bacterial Wilt Caused by R. solanacearum in Chili

#### 3.3.1. Peroxidase Activity (PO)

Leaves

**Figure 5:**
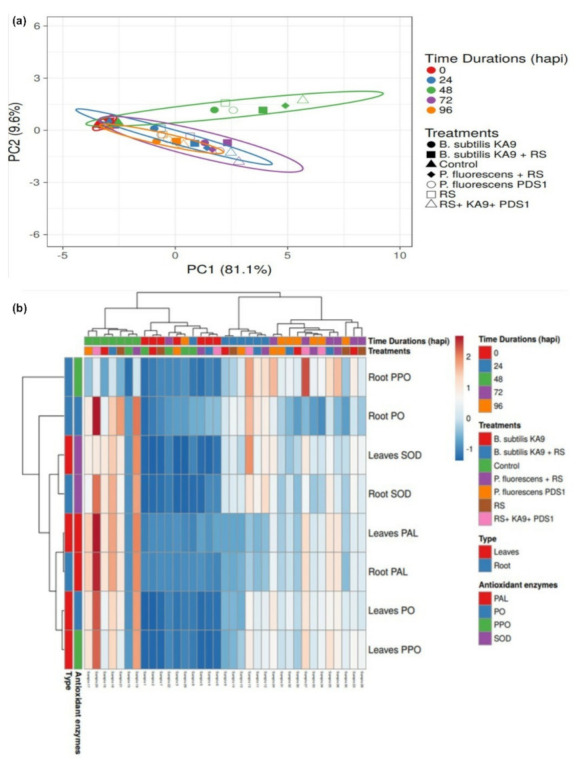
(**a**). Principal component analysis of effect of volatile compounds produced by biocontrol agents on the induction of defensive enzyme activities in chili leaves and roots against *R. solanacearum* in glasshouse conditions. (**b**) Heatmap showing the effect of volatile compounds produced by biocontrol agents on the induction of PAL, PO, PPO and SOD activities in chili leaves and roots against *R. solanacearum* at different time intervals in glasshouse conditions.

Peroxidase activity was measured as the change in OD per minute per milligram of protein. The maximum PO activity was observed in treatment T6 (KA9 + PDS1 + RS) (0.090 ± 0.002 ΔOD·min^−1^·mg^−1^ protein) followed by T5 (0.080 ± 0.002 ΔOD·min^−1^·mg^−1^ protein) at 48 hapi in leaf tissue. At 24 h after pathogen inoculation, the maximum PO activity was observed in T6 (0.056 ± 0.003 ΔOD·min^−1^·mg^−1^ protein) followed by T5 (0.049 ± 0.002 ΔOD·min^−1^·mg^−1^ protein) and T4 (0.046 ± 0.003 ΔOD·min^−1^·mg^−1^ protein) as compared to the pathogen-treated plant (0.029 ± 0.002 ΔOD·min^−1^·mg^−1^ protein). At 72 hapi, the maximum PO activity was observed in T6 (0.064 ± 0.002 ΔOD·min^−1^·mg^−1^ protein) followed by T5 (0.058 ± 0.002 ΔOD·min^−1^·mg^−1^ protein) and T3 (0.061 ± 0.002 ΔOD·min^−1^·mg^−1^ protein) as compared to the pathogen-treated plant (0.043 ± 0.002 ΔOD·min^−1^·mg^−1^ protein) (Supplementary Table S1 and Figure 5b).

The PO activity was increased in all treatments until 48 hapi, after which it declined significantly (Supplementary Table S1). Plants treated with rhizobacteria had higher PO activity than untreated plants (Figure 5b). Furthermore, the *R. solanacearum*-treated plants had considerably higher PO activity at 48 h, i.e., 0.059 ± 0.002, than the untreated plants. However, *P. fluorescens* PDS1 produced volatile compounds that induced higher PO activity than *B*. *subtilis* KA9.

Roots

PO activity is expressed as the unit change in absorbance per minute per milligram of protein (ΔOD·min^−1^·mg^−1^ protein). PO activity in roots expressed similar trends to those seen in leaves, with the maximum activity observed in T6 (0.0092 ± 0.002 ΔOD·min^−1^·mg^−1^ protein) followed by T5 (0.0082 ± 0.002 ΔOD·min^−1^·mg^−1^ protein). At 24 h after pathogen inoculation, the maximum PO activity was observed in T6 (0.0073 ± 0.021 ΔOD·min^−1^·mg^−1^ protein) followed by T4 (0.0060 ± 0.027 ΔOD·min^−1^·mg^−1^ protein) as compared to the pathogen-treated control (0.0052 ± 0.027 ΔOD·min^−1^·mg^−1^ protein). At 48 hapi, the maximum PO activity was observed in T6 (0.0092 ± 0.020 ΔOD·min^−1^·mg^−1^ protein) followed by T5 (0.0082 ± 0.020 ΔOD·min^−1^·mg^−1^ protein) as compared to the pathogen-treated control (0.0077 ± 0.020 ΔOD·min^−1^·mg^−1^ protein). At 72 hapi, the maximum PO activity was observed in T3 (0.0050 ± 0.031 ΔOD·min^−1^·mg^−1^ protein) followed by T4 (0.0043 ± 0.039 ΔOD·min^−1^·mg^−1^ protein) as compared to the pathogen-treated control (0.0036 ± 0.021 ΔOD·min^−1^·mg^−1^ protein) (Supplementary Table S2 and Figure 5b).

The PO activity in chili roots increased for 48 h following pathogen inoculation, before decreasing significantly (Figure 5). Only plants treated with rhizobacteria had higher PO activity than untreated plants (Supplementary Table S2). Furthermore, plants treated with *R. solanacearum* only showed an increase in PO activity after 48 h, i.e., 0.0077 ± 0.020 ΔOD·min^−1^·mg^−1^ protein, which was substantially higher than in untreated plants. PO activity was more significant in *P. fluorescens* PDS1 than in *B. subtilis* KA9. According to the principal component analysis in glasshouse conditions, the effects of volatile compounds produced by biocontrol agents on the induction of peroxidase (PO) activity in chili leaves and roots against *Ralstonia solanacearum* were greatest for treatment T6 at 48 h (0.055 and 0.051, respectively). However, the induction of this defense enzyme was slightly higher in leaves (1.07-fold) than in roots (Figure 5a).

#### 3.3.2. Polyphenol Oxidase Activity (PPO)

Leaves

The polyphenol oxidase activity was measured as the change in absorbance per minute per milligram of protein (ΔOD·min^−1^·mg^−1^ protein). The maximum PPO activity at 24 hapi was observed in T6 (0.055 ± 0.001 ΔOD·min^−1^·mg^−1^ protein) followed by T5 (0.047 ± 0.001ΔOD·min^−1^·mg^−1^ protein) as compared to the pathogen-treated plant (0.027 ± 0.001 ΔOD·min^−1^·mg^−1^ protein). At 48 hapi, the maximum PPO activity was observed in T6 (0.082 ± 0.001 ΔOD·min^−1^·mg^−1^ protein) followed by T5 (0.074 ± 0.001 ΔOD·min^−1^·mg^−1^ protein). At 72 hapi, the maximum PPO activity was observed in T6 (0.064 ± 0.003 ΔOD·min^−1^·mg^−1^ protein) and T4 (0.060 ± 0.003 ΔOD·min^−1^·mg^−1^ protein) as compared to the pathogen-treated plant (0.049 ± 0.002 ΔOD·min^−1^·mg^−1^ protein) (Supplementary Table S3 and Figure 5b).

PPO activity increased in all treatments until 48 h after pathogen inoculation, before decreasing significantly, except for the pathogenic treatment T7 (Figure 5b). Plants treated with rhizobacteria had higher PPO activity than untreated plants (Supplementary Table S3). Furthermore, only *R. solanacearum*-treated plants showed a substantial increase in PPO activity at 72 hapi, i.e., 0.049 ± 0.002, compared to untreated plants. PPO activity was more increased by volatile molecules generated by *P. fluorescens* PDS1 than by *B. subtilis* KA9.

Roots

Polyphenol oxidase activity was measured as the change in absorbance per minute per milligram of protein (ΔOD·min^−1^·mg^−1^ protein). At 72 h after pathogen inoculation, treatment T6 (0.0075 ± 0.034 ΔOD·min^−1^·mg^−1^ protein) had the highest PPO activity in root tissues, followed by T3 (0.0064 ± 0.033 ΔOD·min^−1^·mg^−1^ protein). The maximum PPO activity was observed in T6, where volatile compounds produced by two rhizobacteria were inoculated (0.0067 ± 0.030 ΔOD·min^−1^·mg^−1^ protein) followed by T5 (0.0058 ± 0.032 ΔOD·min^−1^·mg^−1^ protein) as compared to the pathogen-treated plant (0.0035 ± 0.026 ΔOD·min^−1^·mg^−1^ protein) at 24 hapi. The maximum PPO activity was observed in T6 (0.0043 ± 0.028 ΔOD·min^−1^·mg^−1^ protein) followed by T5 (0.0038 ± 0.031 ΔOD·min^−1^·mg^−1^ protein) and T4 (0.0038 ± 0.027 ΔOD·min^−1^·mg^−1^ protein) (Supplementary Table S4 and Figure 5b). The PPO activity in chili roots was increased in all treatments until 72 h after pathogen inoculation, before declining significantly (Figure 5b). The PPO activity due to the production of VOCs by rhizobacteria-treated plants was higher than that of untreated plants (Supplementary Table S4). Furthermore, plants treated with *R. solanacearum* only showed an increase in PPO activity after 72 h, with 0.0042 ± 0.020 ΔOD·min^−1^·mg^−1^ protein, which was considerably greater than that in untreated plants. PO activity was greater in *P. fluorescens* PDS1 than in *B. subtilis* KA9. In glasshouse conditions, a mean value comparison on the effect of volatile compounds produced by biocontrol agents under glasshouse conditions, treatment T6 recorded the maximum polyphenol oxidase (PPO) enzyme activities in roots and leaves of chilli against *R*. *solanacearum* with 0.0050 and 0.052 respectively. This defense related enzyme was activated 10.40-fold more in the leaves than the roots (Figure 5a).

#### 3.3.3. Superoxide Dismutase (SOD)

Leaves

The activity of superoxide dismutase was measured as the change in absorbance per minute per milligram of protein. When compared to untreated healthy plants, SOD activity increased in all treatments in the current study. At 24 hapi, the maximum SOD activity was observed due to the volatile compounds produced by rhizobacteria in T6 (0.081 ± 0.002 ΔOD·min^−1^·mg^−1^ protein) followed by T5 (0.062 ± 0.003 ΔOD·min^−1^·mg^−1^ protein) as compared to the pathogen-treated plant (0.051 ± 0.002 ΔOD·min^−1^·mg^−1^ protein). At 48 hapi, the maximum SOD activity was observed in T5 (0.082 ± 0.004 ΔOD·min^−1^·mg^−1^ protein) followed by T4 (0.072 ± 0.002 ΔOD·min^−1^·mg^−1^ protein). At 72 hapi, the maximum SOD activity was observed in T6 (0.063 ± 0.003 ΔOD·min^−1^·mg^−1^ protein) followed by T3 (0.062 ± 0.003 ΔOD·min^−1^·mg^−1^ protein) as compared to the pathogen-treated control (0.056 ± 0.004 ΔOD·min^−1^·mg^−1^ protein) (Supplementary Table S5 and Figure 5b).

SOD activity was enhanced in all treatments until 48 h after pathogen inoculation, except T6, before considerably decreasing (Figure 5b). SOD activity was higher in rhizobacteria-treated plants than in untreated plants (Supplementary Table S5). Furthermore, plants treated with only *R. solanacearum* showed an increase in SOD activity after 48 h, 0.063 ± 0.003, which was substantially higher than in untreated plants. The volatile compounds generated *by B. subtilis* KA9 treatment increased SOD activity more than those generated by *P. fluorescens* PDS1.

Roots

Maximum SOD activity at 24 hapi was observed due to the volatile compounds produced by rhizobacteria in T6 (0.0091 ± 0.026 ΔOD·min^−1^·mg^−1^ protein) followed by T5 (0.0085 ± 0.025 ΔOD·min^−1^·mg^−1^ protein) in the roots of chili plant. The maximum SOD activity at 48 hapi was observed in T5 (0.0075 ± 0.029 ΔOD·min^−1^·mg^−1^ protein) followed by T6 (0.0072 ± 0.031 ΔOD·min^−1^·mg^−1^ protein) as compared to the pathogen-treated control (0.0056 ± 0.032 ΔOD·min^−1^·mg^−1^ protein). At 72 hapi, the maximum SOD activity was observed in T4 (0.0068 ± 0.030 ΔOD·min^−1^·mg^−1^ protein) followed by T2 (0.0064 ± 0.031 ΔOD·min^−1^·mg^−1^ protein) as compared to the pathogen-treated plant (0.0056 ± 0.031 ΔOD·min^−1^·mg^−1^ protein) (Supplementary Table S6 and Figure 5b).

**Figure 6:**
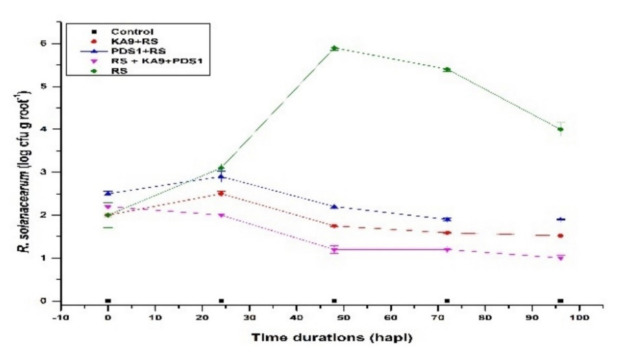
Population of *R. solanacearum* in the rhizospheric zone of the soil with chili plants under different treatments in glasshouse conditions.

The SOD activity in chili roots increased until 28 h after pathogen inoculation in all treatments, before decreasing dramatically (Figure 6). Dual rhizobacteria-treated plants had higher SOD activity than single-rhizobacteria-treated and untreated plants (Supplementary Table S6). Furthermore, plants treated with only *R. solanacearum* showed a substantial increase in SOD activity after 48 h, i.e., 0.0065 ± 0.026 ΔOD·min^−1^·mg^−1^ protein, compared to untreated plants. However, there was no significant difference between *P. fluorescens* PDS1 and *B*. *subtilis* KA9 in terms of the induction of SOD enzyme activity in the root tissue of chili plants.

A mean value comparison on the effect of volatile compounds produced by biocontrol agents under glasshouse conditions, treatment T6 recorded the maximum SOD enzyme activities in roots and leaves of chilli against *R*. *solanacearum* with 0.0057 and 0.052 respectively. This defense related enzyme was activated 9.12-fold -fold more in the leaves than the roots (Figure 5a).

#### 3.3.4. Phenyl Ammonia Lyase (PAL)

Leaves

Phenyl ammonia lyase activity is expressed in µmol *t*-cinnamate·min^−1^·g^−1^ FW. The present study found that PAL activity increased in all treatments, compared to unchallenged untreated healthy control plants. Overall, maximum PAL activity was observed due to volatile compounds produced by rhizobacteria in T6 (312.667 ± 2.64 µmol *t*-cinnamate·min^−1^·g^−1^ FW) followed by T5 (265.00 ± 1.64 µmol *t*-cinnamate·min^−1^·g^−1^ FW) at 48 hapi. At 24 hapi, the maximum PAL activity was observed in T6 (88.33 ± 3.24 µmol·min^−1^·g^−1^ FW) and T5 (84.333 ± 1.27 µmol·min^−1^·g^−1^ FW) as compared to the pathogen-treated control (63.666 ± 2.25 µmol·min^−1^·g^−1^ FW). At 72 hapi, the maximum PAL activity was observed in T6 (207.000 ± 3.12 µmol·min^−1^·g^−1^ FW) followed by T5 (192.00 ± 1.64 µmol·min^−1^·g^−1^ FW) and T4 (191.933 ± 2.34 µmol·min^−1^·g^−1^ FW) as compared to the pathogen-treated plant (134.333 ± 2.21 µmol·min^−1^·g^−1^ FW) (Supplementary Table S7 and Figure 5b). The PAL activity was increased in all treatments up to 48 hapi, after which it declined significantly (Figure 5a). The PAL activity due to the volatile compounds produced by rhizobacteria-treated plants was higher than that in untreated plants (Supplementary Table S7). After 48 h, *R*. *solanacearum*-treated plants exhibited a significantly increased in PAL activity (188.667 ± 3.24) in comparison to the untreated plants. However, the volatile produced by *P. fluorescens* PDS1 enhanced the induction of PAL activity in the leaves of chilli cv. PusaJwala as compared to *B*. *subtilis* KA9. 

Roots

Overall, the maximum PAL activity was observed in treatment T6 (299.67 ± 3.40 µmol *t*-cinnamate·min^−1^·g^−1^ FW) followed by T5 (254.67 ± 3.16 µmol *t*-cinnamate·min^−1^·g^−1^ FW) at 48 hapi in roots of chili cv. Pusa Jwala. At 24 hapi, the maximum PAL activity was observed in T6 (88.00 ± 2.00 µmol·min^−1^·g^−1^ FW) and T5 (80.33 ± 2.17 µmol·min^−1^·g^−1^ FW) as compared to the pathogen-treated plant (58.00 ± 2.86 µmol·min^−1^·g^−1^ FW). At 72 hapi, the maximum PAL activity was observed in T6 (199.33 ± 3.42 µmol·min^−1^·g^−1^ FW), as well as T5 (187.00 ± 2.24 µmol·min^−1^·g^−1^ FW) and T4 (185.33 ± 2.84 µmol·min^−1^·g^−1^ FW), followed by T3 (141.67 ± 2.73 µmol·min^−1^·g^−1^ FW) as compared to the pathogen-treated control (124.33 ± 2.92 µmol·min^−1^·g^−1^ FW) (Supplementary Table S8 and Figure 5b).

The PAL activity in chili roots was increased in all treatments up to 48 hapi, after which it declined significantly (Figure 5b). Volatile compounds produced by dual-rhizobacteria-treated plants showed more PAL activity than those produced by individual rhizobacteria-treated plants and untreated plants (Supplementary Table S8). Furthermore, after 48 hapi, *R. solanacearum*-treated plants also showed an increase in PAL activity (184.67 ± 2.65 µmol·min^−1^·g^−1^ FW), which was substantially higher than in untreated plants. Both rhizobacteria produced volatile compounds with potential for inducing PAL enzyme activation. *P. fluorescens* PDS1, on the other hand, triggered greater PAL activity than *B. subtilis* KA9. A mean value comparison on the effect of volatile compounds produced by biocontrol agents under glasshouse conditions, treatment T6 recorded the maximum PAL enzyme activities in roots and leaves of chilli against *R*. *solanacearum* with 149.80 and 145.80 respectively. This defene related enzyme was activated 1.027-fold more in the root tissues than the leaves (Figure 5a).

### 3.4. Colonization Behavior of R. solanacearum in the Rhizospheric Zone of the Soil with Chili Plants

Populations of *R. solanacearum* in the rhizospheric zone of the soil were detected using the plate counting method. The population of *R. solanacearum* increased rapidly after inoculation and reached a maximum on 48 h (5.9 ± 0.06 logCFU·g*^−^*^1^ root) in the RS treatment, which was significantly greater than in the uninoculated control. A significant low population of *R. solanacearum* was observed in the treatments where rhizobacteria were applied with *R. solanacearum*. As the time period increased, there was decline in the microbial population (Figure 6, Supplementary Table S12).

These overall results indicate that *B*. *subtilis* KA9 and *P. fluorescens* PDS1 effectively suppressed the population of *R. solanacearum* and controlled the chili bacterial wilt caused by *R. solanacearum*.

### 3.5. GC–MS Analysis of Volatile Compounds Present in Different Treatments of Rhizobacteria

**Figure 7:**
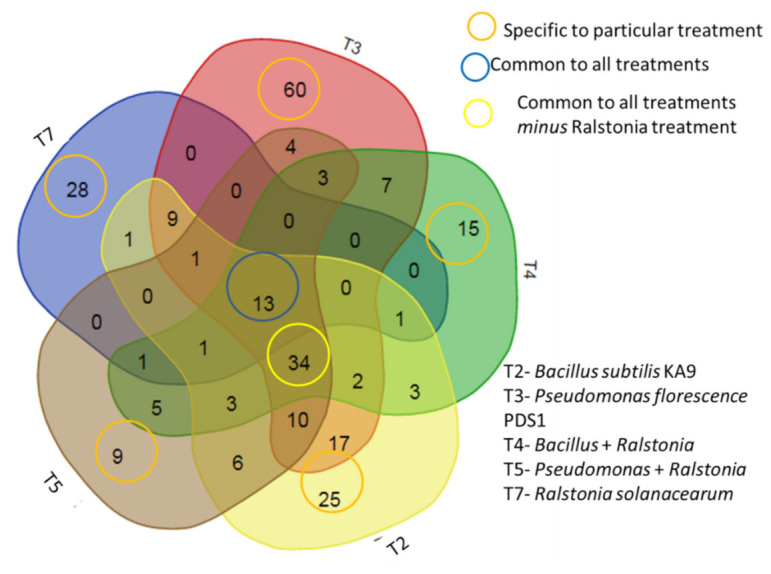
Venn diagram showing total number of volatile compounds produced by rhizobacteria and *R. solanacearum* during the induction of defensive enzymes against bacterial wilt caused by *R. solanacearum* in chili.

The separation and identification of volatile compounds from the plastic bottle assembly were achieved using a Shimadzu QP 2000 fitted with an Rtx-5 ms column (30 m × 0.25 mm) packed with 95% dimethyl poly siloxane for GC–MS analysis. The mass spectra of the unknown compounds were compared with those of known compounds stored in the NIST-011 library. The active constituents, along with their retention time (RT), molecular weight, molecular formulae, and concentration (peak area%), were determined from the five different rhizobacteria treatments, namely, T2 (*B*. *subtilis* KA9), T3 (*P. fluorescens* PDS1), T4 (*B*. *subtilis* KA9 + *R. solanacearum*), T5 (*P. fluorescens* + *R. solanacearum*), and T7 (*R. solanacearum*). Samples were collected from these treatments for GC–MS analysis, which revealed the presence of 257 compounds among all samples. A total of 137 molecules were specifically present in certain treatments, whereas the remaining 120 molecules were common to multiple samples. Furthermore, 13 compounds were common to all treatments (Figure 7, Table 2). Common compounds with their retention time (RT) included azulene (13.64), 1,2,3-trimethyl benzene (10.045), cycloheptasiloxane (15.6–20.13), hexamethyl-cyclotrisiloxane (6.095), dodecane (13.74), ethylbenzene (7.05), tetradecane (16.715), undecane (12.035), and other benzene derivatives such as toluene, as well as various hydrocarbons in the form of alkanes and silicone derivatives. Most of these compounds were aromatic in nature, emphasizing the basic metabolic process of the microbes.

By analyzing the mass spectra to most in the NIST Mass Spectral Library (probability-based similarity >85 percent), the chemicals likely produced by various rhizobacterial treatments of *B. subtilis* KA9 and *P. fluorescens* PDS1 was identified. The peak area of each compound was calculated as a percentage relative to the total peak area of all volatile organic compounds in a particular treatment. Major compounds such as 2,6-dimethylpyrazine (20.8), 2-methyl-1-butanol (14.3), 4-ethylbenzoic acid, 2-bromo-4-fluorophenyl (17.49), anisole (12), benzaldehyde, 3,5-dimethyl- (13.51), benzothiazole (15.00), butylthiophenol (11.9), methyl butanal (8.2), octanol (12.5), and oxime-, methoxy-phenyl- (8.195) were identified (Table 3, Supplementary Figure S3a,b,d,e).

The gas chromatography–mass spectrometry (GC–MS) profile of common volatile organic compounds (VOCs) produced by the treatment (T7) with *R. Solanacearum* revealed benzene derivatives, different hydrocarbons in the form of alkanes and esters, and carboxylic acids. Most compounds were aromatic in nature, emphasizing the basic metabolic process of *R. solanacearum* (Table 4, Supplementary Figure S3c).

### 3.6. Expression of Rhizobacteria-Mediated Defense-Related Genes against R. solanacearum in Chili

**Figure 8:**
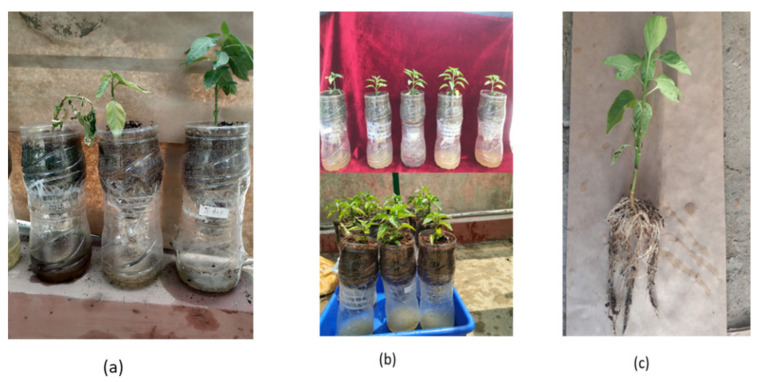
Effect of volatile compounds produced by rhizobacteria on the induction of defensive enzymes against bacterial wilt caused by *R. solanacearum* in chili in glasshouse conditions: (**a**) wilted and healthy plants; (**b**) plastic bottle assembly used to study volatile compounds; (**c**) chili plant used for growth parameter observations.

A total of 12 genes, namely, PAL, POD, SOD, WRKYa, PAL1, DEF-1, CAT-2, WRKY40, HSFC1, LOX2, and NPR1, along with the transcript abundance of ubiquitin E3 as an endogenous control, were chosen to analyze the expression of defense-related genes in the leaf and root tissues of chili. The inoculated tissues of chili plants were harvested at 48 h post inoculation of rhizobacteria (i.e., *Bacillus subtilis* and *Pseudomonas fluorescens*) from the bottom of the apparatus designed to produce volatile compounds that can induce ISR through the root tissues of plants against pathogenic microbe *R. solanacearum* (Figure 8).

#### Gene-Specific Primer Amplification of cDNA

**Figure 9:**
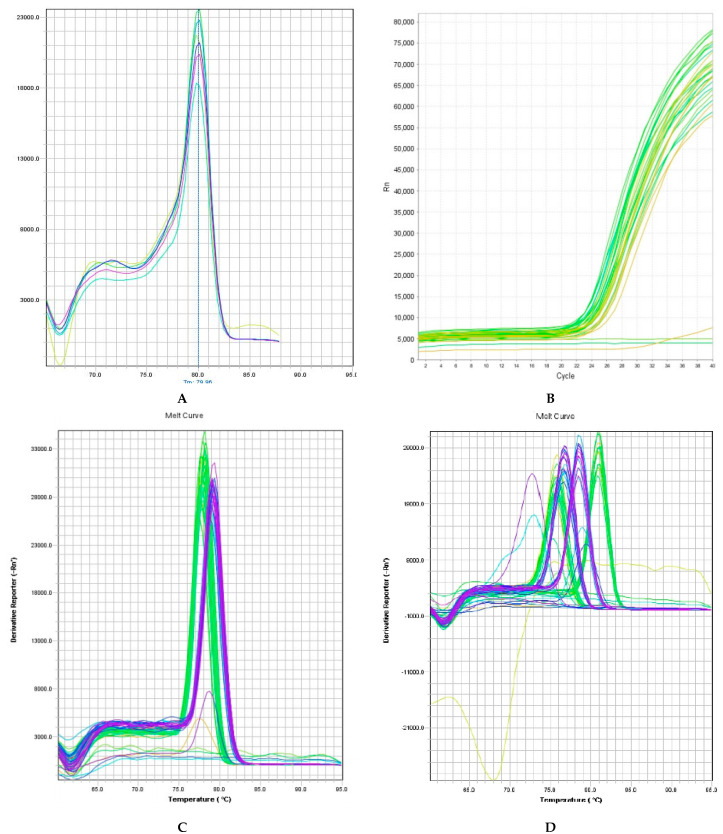
Melting curves observed for qPCR analysis of the relative expression of PAL, POD, SOD, WRKYa, PAL1, DEF-1, CAT-2, WRKY40, HSFC1, LOX2, and NPR1 genes normalized to UBI3 after different treatments: mock, RS (*R. solanacearum*), KA9 + RS (*Bacillus subtilis* + *R. solanacearum*), PDS1 + RS (*Pseudomonas fluorescens* + *R. solanacearum*), KA9 + PDS1 + RS (*Bacillus subtilis* + *Pseudomonas fluoresecens* + *R. solanacearum*), and BABA (chemical control). Melting curve (**A**) and amplification plot (**B**) observed for qPCR analysis of relative expression of normalized Ubi3 gene in chili. (**C**) Genes yielding a single melting curve. (**D**) Genes shown multiple peaks (marked by red arrows) were excluded from the analysis.

The RT-PCR assay was performed using three replicates of each sample, for which cDNA synthesis was performed separately and independently. Post PCR, both the amplification plot and the melting curve plot were examined for all 12 genes (Figure 9a–d). It can be observed that most genes yielded a single melting curve; those yielding multiple peaks (marked by red arrows) were excluded from the analysis. Ubi3 gave a single peak at ~80 °C, suggesting the cDNA to be of good quality. Similarly, the sigmoidal curve obtained in the amplification plot indicated ideal PCR amplification efficiency as single melting curves were observed for various defensive genes. The relative abundance of the transcripts was analyzed by normalizing the transcript abundance data to *Ubi3*, followed by calibration.

The expression of lipoxygenase (LOX), peroxidase (POD), phenyl ammonia lyase (PAL), superoxide dismutase (SOD), catalase (CAT), and defensin gene (DEF) is known to directly reflect ISR at both the transcript and the protein levels. Other genes such as WRKY (WRKYa, WRKY40) and HSF (HSFC) are transcription factors that act as activators or repressors of plant immunity. These genes are also implicated in pathogen-associated molecular pattern-triggered immunity and effector-triggered immunity by influencing gene expression. Furthermore, NPR1 (a nonexpressor of pathogenesis-related genes) contributes to overall plant health when the immune response of the plant is increased. Its expression does not change dramatically upon pathogen infection; hence, its post-translational modifications are essential. It acts as a transcription cofactor, whose expression changes in leaves because of root-associated bacteria.

### 3.7. Relative Expression of Defense-Related Genes in the Roots and Leaves of Chili against R. solanacearum in Glasshouse Conditions

#### 3.7.1. PAL (Phenyl Ammonia Lyase) Gene

**Figure 10:**
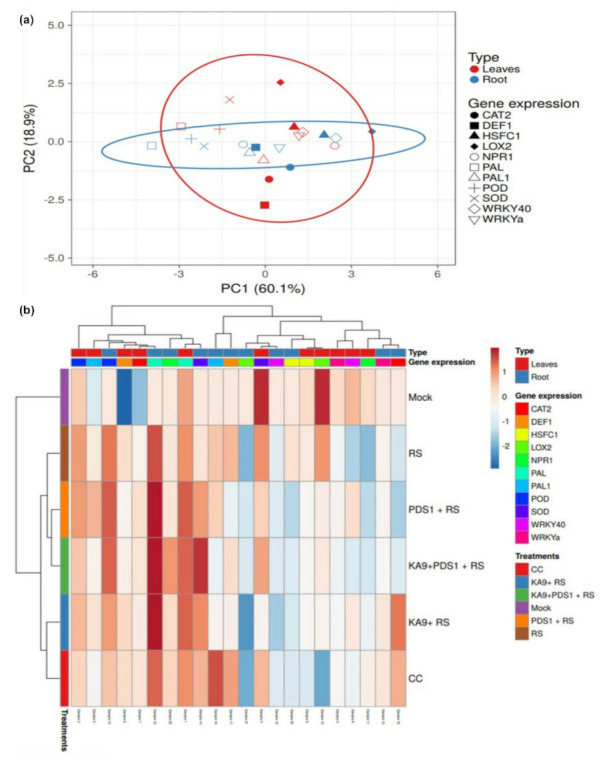
(**a**) Principal component analysis of defense-related genes in terms of expression levels following treatment with biocontrol agents in leaves and roots of chili against *R. solanacearum* in glasshouse conditions (**b**) Elicitation of defense-related gene expression (PAL, POD, SOD, WRKYa, PAL1, DEF-1, CAT-2, WRKY40, HSFC1, LOX2, and NPR1) by *P. fluorescens* PDS1 and *B. subtilis* KA9 in chili against *R. solanacearum* pathogen challenge in roots and leaves, 48 h after inoculation in glasshouse conditions.

Ubiquitin 3 was used as an internal control to normalize the reaction, and double delta Ct analysis was conducted to determine the fold change in expression of the gene product; the values were plotted on a graph. The relative expression of PAL in response to different treatments (RS (*R. solanacearum*), KA9 + RS (*B. subtilis* KA9 + *R. solanacearum*), PDS1 + RS (*P. fluorescens* PDS1 + *R. solanacearum*), KA9 + PDS1 + RS (*B. subtilis* KA9 + *P. fluorescens* PDS1 + *R. solanacearum*), BABA (chemical control), and mock inoculated treatments) on susceptible chili cv. Pusa Jwala is represented graphically. In the susceptible cv. Pusa Jwala, the mock treatment was used for calibration, revealing that the BABA treatment at 1000 µg/mL resulted in the highest relative expression of PAL (4.48-fold) at 48 h, followed by KA9 + PDS1 + RS (3.75-fold), KA9 + RS (3.25-fold), PDS1 + RS (3.09-fold), and RS (2.54-fold). These was no significant variation observed in relative expression in response to PDS1 + RS and KA9 + RS treatments. Moreover, the dual rhizobacterial inoculation treatment, i.e., KA9 + PDS1 + RS, led to a significant variation compared to other treatments. Upregulation of defensive gene PAL was found in all treatments as compared to UBI3 (Figure 10a,b) (Supplementary Table S9).

In leaves, the maximum relative expression of PAL gene was recorded at 48 h after treatment with BABA (4.28-fold), followed by KA9 + PDS1 + RS (3.25-fold), KA9 + RS (2.75-fold), PDS1 + RS (2.59-fold), and RS (2.04-fold) as compared to UBI3. A significant variation was observed in the dual KA9 + PDS1 + RS treatment compared to the mock inoculated control, as well as individual rhizobacterial treatments. Upregulation of defensive gene PAL in leaves was found in all treatments as compared to UBI3 (Supplementary Table S10). The relative expression of PAL gene in root samples was greater than in leaf samples after all treatments.

#### 3.7.2. POD (Peroxidase) Gene

In the susceptible cv. Pusa Jwala, the mock treatment was used to calibrate the expression of defense-related genes. The highest relative expression of POD gene was recorded at 48 h after treatment with BABA (3.5-fold), followed by KA9 + PDS1 + RS (3.22-fold), PDS1 + RS (2.6-fold), KA9 + RS (2.29-fold), and RS (2.26-fold). The relative expression observed in response to RS and KA9 + RS treatments was not significantly different during the study. However, the dual rhizobacterial treatment KA9 + PDS1 + RS was found to vary significantly from individual rhizobacterial inoculated treatments. Upregulation of defensive gene POD was found in all treatments as compared to UBI3 in the plant roots (Figure 10a,b) (Supplementary Table S9). In leaves, a similar pattern of POD gene expression was recorded. The highest relative expression of POD in leaf samples of cv. Pusa Jwala was recorded at 48 h after treatment with BABA (3.15-fold), followed by KA9 + PDS1 + RS (2.39-fold), PDS1 + RS (2.10-fold), KA9 + RS (1.99-fold), and RS (1.96-fold). A significant variation was observed in the relative expression after dual rhizobacterial treatment (KA9 + PDS1 + RS) compared to individual rhizobacterial treatments, but no significant variation in relative expression was observed between PDS1 + RS and KA9 + RS treatments. Upregulation of defensive gene POD in leaves was found in all treatments as compared to UBI3 (Supplementary Table S10). The relative expression of POD gene in root samples of cv. Pusa Jwala was greater than in leaf samples after all treatments in glasshouse conditions.

#### 3.7.3. SOD (Superoxide Dismutase) Gene

The highest relative expression of SOD gene was recorded at 48 h in roots of cv. Pusa Jwala after treatment with BABA (3.68-fold), followed by KA9 + PDS1 + RS (3.57-fold), KA9 + RS (2.46-fold), PDS1 + RS (2.12-fold), and RS (1.03-fold). The relative expression observed in response to PDS1 + RS and KA9 + RS treatments was not significantly different; however, there was a significant variation between dual rhizobacterial treatment (KA9 + PDS1 + RS) and individual rhizobacterial treatments after 48 h. Upregulation of defensive gene SOD was found in all treatments compared to UBI3. In leaves of cv. Pusa Jwala, the highest relative expression of SOD was recorded at 48 h after treatment with BABA (3.60-fold), followed by KA9 + PDS1 + RS (2.70-fold), PDS1 + RS (1.62-fold), KA9 + RS (1.00-fold), and RS (2.03-fold). The relative expression observed in the dual rhizobacterial inoculation treatment (KA9 + PDS1 + RS) was significantly different from that in individual rhizobacterial treatments. The relative expression observed in response to control and KA9 + RS treatments was not significantly different after 48 h inoculation of rhizobacteria. Upregulation of defensive gene SOD in leaves was found in all treatments compared to UBI3 (Figure 10a,b) (Supplementary Tables S9 and S10). The relative expression of SOD gene in root samples of cv. Pusa Jwala was greater than in leaf samples after all treatments.

#### 3.7.4. WRKYa Gene

The highest relative expression of WRKYa gene in roots was recorded at 48 h after treatment with BABA (2.66-fold), followed by KA9 + PDS1 + RS (1.43-fold), KA9 + RS (1.36-fold), PDS1 + RS (0.82-fold), and RS (0.64-fold). No significant variation in the relative expression of WRKYa gene was recorded in roots of cv. Pusa Jwala between dual rhizobacterial treatment (KA9 + PDS1 + RS) and individual rhizobacterial treatments. Upregulation of defensive gene WRKYa was found in all treatments compared to UBI3. In leaves, the highest relative expression of WRKYa was recorded at 48 h after treatment with BABA (1.90-fold), followed by KA9 + PDS1 + RS (1.20-fold), PDS1 + RS (0.82-fold), KA9 + RS (0.80-fold), and RS (0.32-fold). A significant variation in relative expression between dual rhizobacterial treatment (KA9 + PDS1 + RS) and individual rhizobacterial treatments was recorded in leaf samples after 48 h of treatment. However, the relative expression observed in response to KA9 + RS and PDS 1 + RS treatments was not significantly different. Upregulation of defensive gene WRKYa in leaves was found in all treatments compared to UBI3 (Figure 10 WRKY-Leaf). The relative expression of WRKYa gene in the roots of cv. Pusa Jwala was greater than leaf samples after all treatments (Figure 10a,b) (Supplementary Tables S9 and S10).

#### 3.7.5. PAL1 (Phenyl Ammonia Lyase) Gene

The highest relative expression of PAL1 gene was recorded in the roots of cv. Pusa Jwala at 48 h after treatment with BABA (5.04-fold), followed by PDS1 + RS (1.58-fold), RS (1.51-fold), KA9 + PDS1 + RS (1.17-fold), and KA9 + RS (0.99-fold). The relative expression observed after PDS1 + RS treatment was significantly different from the control and other treatments. Upregulation of defensive gene PAL1 was found in all treatments compared to UBI3. In leaves, a similar pattern of PAL1 gene expression was recorded. The highest relative expression of PAL1 was recorded at 48 h after treatment with BABA (2.00-fold), followed by PDS1 + RS (1.90-fold), KA9 + PDS1 + RS (1.57-fold), KA9 + RS (1.50-fold), and RS (0.86-fold). The relative expression observed after the PDS1 + RS treatment differed significantly from the mock control and other treatments. Upregulation of defensive gene PAL1 was found in all treatments except for the uninoculated mock, which presented a downregulation compared to UBI3. Rhizobacteria induced PAL1 gene expression more in leaf tissue than in root tissue, whereas chemical BABA treatment induced PAL1 gene expression more in root tissue than in leaf tissue (Figure 10a,b) (Supplementary Tables S9 and S10).

#### 3.7.6. DEF1 (Defensive) Gene

The highest relative expression of DEF1 gene was recorded at 48 h after treatment with BABA (4.03-fold), followed by KA9 + PDS1 + RS (2.11-fold), KA9 + RS (1.11-fold), PDS1 + RS (0.48-fold), and RS (1.62-fold). The combined KA9 + PDS1 + RS treatment showed a significant variation in relative expression compared to other rhizobacterial treatments. Upregulation of defensive gene DEF1 was found in all treatments compared to UBI3. In leaves, the highest relative expression of DEF1 was recorded in cv. Pusa Jwala at 48 h after treatment with BABA (3.00-fold), followed by KA9 + PDS1 + RS (1.61-fold), KA9 + RS (1.70-fold), PDS1 + RS (0.90-fold), and RS (1.20-fold). The dual rhizobacterial treatment (KA9 + PDS1 + RS) differed significantly in terms of the relative expression of DEF1 from individual rhizobacterial treatments. Upregulation of defensive gene DEF1 in leaves was recorded in all cases, except for the uninoculated mock which exhibited a downregulation compared to UBI3. It was also noted that, after RS, KA9 + PDS1 + RS, and BABA treatments, DEF1 gene was expressed more in root tissue than in leaf tissue, whereas the opposite was found for KA9 + RS and PDS1 + RS treatments (Figure 10a,b) (Supplementary Tables S9 and S10).

#### 3.7.7. CAT2 (Catalase 2) Gene

The highest relative expression of CAT2 gene was recorded at 48 h in root tissue of cv. Pusa Jwala after treatment with BABA (3.75-fold), followed by KA9 + PDS1 + RS (0.91-fold) and KA9 + RS (2.6-fold). There was no significant variation between PDS1 + RS (−0.34-fold) and RS (−0.34-fold) treatments; however, both cases exhibited downregulation of CAT2. The relative expression observed in the dual rhizobacterial inoculation (KA9 + PDS1 + RS) differed significantly from individual rhizobacterial treatments. Upregulation of defensive gene CAT2 was found after treatment with BABA, PDS1 + KA9 + RS, and KA9 + RS, whereas downregulation was observed for PDS1 + RS and RS, compared to UBI3. In leaves, the highest relative expression of CAT2 gene was recorded at 48 h after treatment with BABA (2.70-fold), followed by KA9 + PDS1 + RS (1.91-fold), PDS1 + RS (1.34-fold), KA9 + RS (1.20-fold), and RS (0.60-fold). The relative expression of CAT2 gene observed after dual rhizobacterial treatment with KA9 + PDS1 + RS differed significantly from individual rhizobacterial treatments. Upregulation of defensive gene CAT2 was recorded in leaves, except for the uninoculated mock which exhibited downregulation compared to UBI3. KA9 + RS, KA9 + PDS1 + RS, and BABA treatments induced more CAT2 activity in the roots of cv. Pusa Jwala, whereas the opposite was found for RS and PDS1 + RS treatments (Figure 10a,b) (Supplementary Tables S9 and S10).

#### 3.7.8. WRKY40 Gene

The highest relative expression of WRKY40 gene was recorded at 48 h in root tissue of cv. Pusa Jwala after treatment with BABA (1.01-fold), followed by KA9 + PDS1 + RS (0.91-fold), and PDS1 + RS (0.24-fold). The relative expression observed in the dual rhizobacterial inoculation (KA9 + PDS1 + RS) differed significantly from individual rhizobacterial treatments. Upregulation of defensive gene WRKY40 was observed after BABA, PDS1 + KA9 + RS, and PDS1 + RS treatment, whereas downregulation of WRKY40 was observed after KA9 + RS and RS treatment, compared to UBI3. BABA treatment differed significantly from rhizobacterial treatments. In leaves, the highest relative expression of WRKY40 was recorded at 48 h after treatment with BABA (1.50-fold), followed by PDS1 + RS (1.40-fold), KA9 + RS (1.30-fold), KA9 + PDS1 + RS (0.90-fold), and RS (−0.65-fold). The relative expression observed after PDS1 + RS (1.40-fold) and KA9 + RS (1.30-fold) treatments was not significantly different, unlike individual rhizobacterial treatments compared to the dual inoculation (KA9 + PDS1 + RS). Upregulation of defensive gene WRKY40 was recorded in leaves, except for the pathogenic treatment of *R. solanacearum*, which exhibited downregulation compared to UBI3. The WRKY40 gene was relatively expressed more in leaf tissue as compared to root tissue after all treatments (Figure 10a,b) (Supplementary Tables S9 and S10).

#### 3.7.9. HFSC1 Gene

The highest relative expression of HFSC1 gene was recorded in root tissues of cv. Pusa Jwala after treatment with RS (1.08-fold), followed by KA9 + PDS1 + RS (0.90-fold) and BABA (0.87-fold), KA9 + RS (0.24-fold), and PDS1 + RS (−0.25-fold). Plants treated with RS only showed a significantly higher relative expression of HFSC1 gene in root tissues than in rhizobacterial treatments. Upregulation of defensive gene HFSC1 was found in treatments BABA, PDS1 + KA9 + RS, and KA9 + RS, whereas downregulation was observed in treatment PDS1 + RS. In leaves, the highest relative expression of HFSC1 was recorded after treatment with KA9 + PDS1 + RS (1.5-fold), followed by KA9 + RS (0.90-fold) and RS (0.90-fold), BABA (0.87-fold), and PDS1 + RS (0.80-fold). The dual culture of rhizobacteria KA9 + PDS1 + RS exhibited a higher relative expression of HFSC1 gene than single rhizobacterial treatments. Upregulation of defensive gene HFSC1 in leaf tissue was observed in all samples compared to UBI3 (Figure 10a,b) (Supplementary Tables S9 and S10). With the exception of RS treatment, there was a higher relative expression of HFSC1 gene in leaf tissue than in root tissue.

#### 3.7.10. LOX2 (Lipoxygenase 2) Gene

The highest relative expression of LOX2 gene was recorded in root tissue of cv. Pusa Jwala at 48 h after treatment with KA9 + PDS1 + RS (0.84-fold), followed by PDS1 + RS (0.04-fold), CC (−0.63-fold), KA9 + RS (−0.93-fold), and RS (−0.98-fold). There was no significant difference in expression of LOX2 between uninoculated mock and PDS + RS treatments. The relative expression observed in the dual microbial inoculation was found significantly higher compared to the mock control and other treatments. Upregulation of defensive gene LOX2 was found after PDS1 + KA9 + RS and PDS1 + RS treatments, whereas downregulation was observed after KA9 + RS, RS, and BABA treatments, compared to UBI3. In leaves, the highest relative expression of LOX2 was recorded after treatment with RS (1.98-fold), followed by KA9 + PDS1 + RS (1.84-fold) KA9 + RS (1.07-fold), and PDS1 + RS (1.04). The lowest relative expression of LOX2 gene was recorded after treatment with BABA. Dual rhizobacterial treatment exhibited a significantly higher relative expression of LOX2 gene compared to single rhizobacterial treatments. Upregulation of defensive gene LOX2 in leaves was observed in all samples except for the chemical control, compared to UBI3. Except for the chemical treatment, all treatments induced greater LOX1 gene expression in leaf tissue than in root tissue after 48 h of inoculation in glasshouse conditions (Figure 10a,b) (Supplementary Tables S9 and S10).

#### 3.7.11. NPR1 (Nonexpressor of Pathogen-Related) Gene

The highest relative expression of NPR1 gene was recorded at 48 h in the tissue of cv. Pusa Jwala after treatment with BABA (3.13-fold), followed by KA9 + PDS1 + RS (0.96-fold), KA9 + RS (0.94-fold), PDS1 + RS (0.82-fold), and RS (0.52-fold). The relative expressional of NPR1 gene in plants treated with KA9 + PDS1 + RS and KA9 + RS did not differ significantly, whereas treatment with the chemical control BABA differed significantly from the mock control and other treatments. Upregulation of defensive gene NPR1 was found in all treatments compared to UBI3. In leaves, the highest relative expression of NPR1 gene was recorded at 48 h after treatment with BABA (2.15-fold), followed by KA9 + RS (0.67-fold), PDS1 + RS (−0.22-fold), KA9 + PDS1 + RS (−0.74-fold), and RS (−0.93-fold). The relative expression of NPR1 gene observed in BABA-treated plants significantly differed from other treatments. Upregulation of defensive gene NPR1 in leaves was found in BABA and KA9 + RS treatments, whereas downregulation occurred in KA9 + PDS1+ RS (−0.22-fold) and PDS1 + RS (−0.22-fold) treatments, compared to UBI3. The root tissue of cv. Pusa Jwala showed a greater relative expression of NPR2 gene at 48 h than the leaf tissue after all treatments (Figure 10a,b) (Supplementary Tables S9 and S10).

### 3.8. Overall Relative Expression of Defensive Genes in Leaves and Roots of Chili

**Figure 11:**
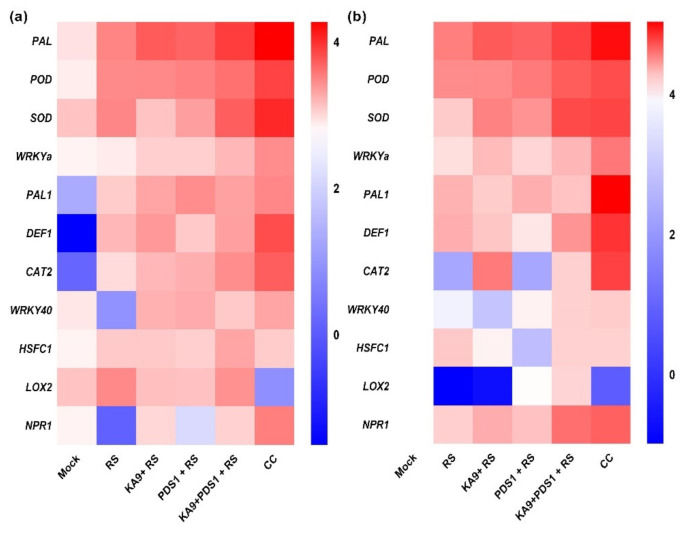
Comparative gene expression (PAL, POD, SOD, WRKYa, PAL1, DEF−1, CAT−2, WRKY40, HSFC1, LOX2, and NPR1 genes) after treatment with *P. fluorescens* PDS1 and *B. Subtilis* KA9 in chili against *R. solanacearum* pathogen challenge in (**a**) leaves and (**b**) roots at 48 h after inoculation in glasshouse conditions.

In this study, the relative expression of 11 defense-related genes was studied in chili cv. Pusa Jwala to determine ISR; in particular, PAL, PAL1, WRKYa, DEF1, CAT2, and WRKY40 genes led to an induction in the plants as compared to the control, with 1–5 log fold changes following exogenous application of BABA (β-aminobutyric acid). It was observed that most genes showed an enhancement of their transcription levels ranging from 0.5–4.0 log fold change. Maximum expression modulation was observed for PAL gene, i.e., ~4.0 log fold increase in both root and leaf tissues. POD, SOD, and CAT2 showed a 3.0 log fold increase after *B. subtilis* KA9 and *P. fluorescens* PDS1 treatments. In contrast, NPR1 does not show an increase after treatment with individual rhizobacteria, but showed a 1.5 log fold increase after all combined treatments except for *Pseudomonas* and *Ralstonia* (Figure 11).

### 3.9. Biocontrol of Bacterial Wilt Disease and Plant Growth Attributes after Different Treatments with Volatile Compound-Producing Rhizobacteria

In the present study, two rhizobacteria *B. subtilis* KA9 and *P. fluorescens* PDS1 were selected on the basis of their antagonistic and plant-growth promoting abilities under in vitro conditions. In glasshouse conditions at the National Phytotron Facility, IARI, New Delhi, these rhizobacteria isolates (KA9 and PDS1) were investigated for their bio-efficacy in managing bacterial wilt disease and growth promotion activities in chili cv. Pusa Jwala (susceptible cv.). Individual and dual inoculation of isolates PDS1 and KA9 significantly reduced the bacterial wilt incidence in chili and enhanced the growth of the plants. Minimum disease intensity (21.80%) was recorded in BABA-treated plants followed by treatment with KA9 + PDS1 + RS (22.40%), KA9 + RS (26.50), and PDS1 + RS (26.80) after 40 days of pathogen inoculation.

**Figure 12:**
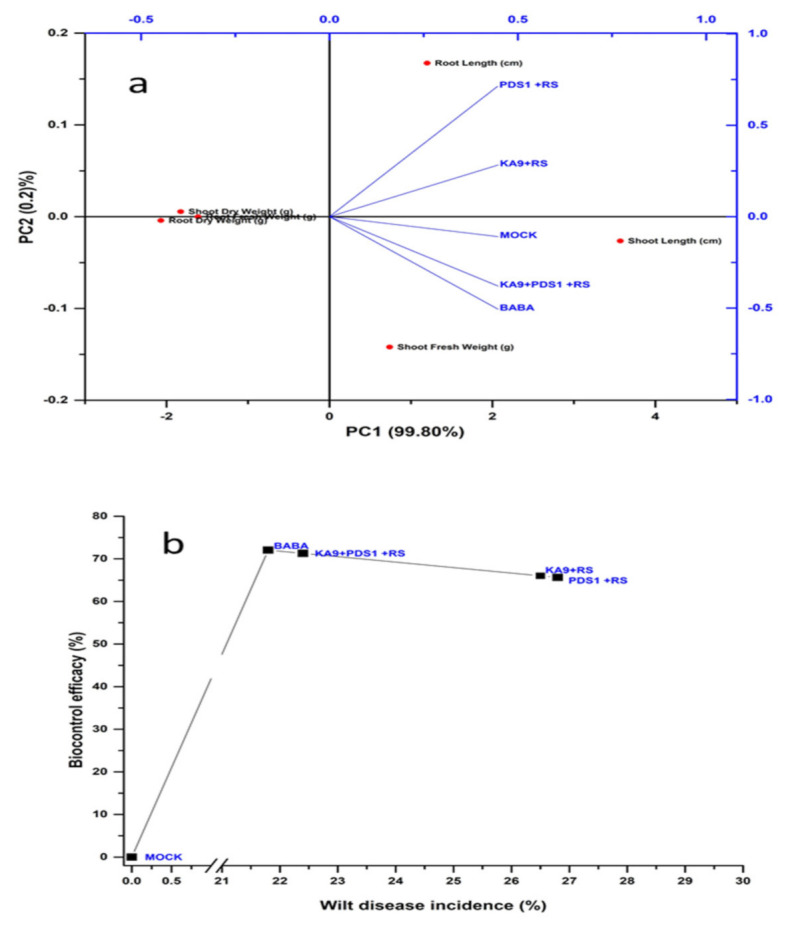
(**a**) Principal component analysis showing correlation and significance of growth metrics impacted by rhizobacterial strains in chili. (**b**) Graph showing biocontrol efficacy (%) following the various treatments with rhizobacteria against bacterial wilt disease.

Maximum biocontrol efficacy (72.05%) was recorded in BABA-treated plants followed by treatment with KA9 + PDS1 + RS (71.28%), KA9 + RS (66.02%), and PDS1 + RS (65.64%). The bacterial wilt disease symptoms were initiated only in *R. solanacearum-*infected plants after 16 days of inoculation, whereas in rhizobacteria-treated plants, appearance of wilt disease was delayed by 10–14 days. Moreover, a significant variation in reduction in wilt disease in chili was recorded among these isolates in glasshouse conditions (*p* > 0.05) (Table 5). After 40 days of treatment, plant growth promoters were documented for their effects on growth promoting efficacy, plant length, root and shoot dry weight. The maximum shoot length (21.70 cm) was recorded after KA9 + PDS1 + RS treatment, followed by BABA (20.8 cm), KA-9 + RS (19.80 cm), PDS1 + RS (18.60 cm), and mock (16.6 cm), while the minimum shoot length was recorded after RS treatment (10.30 cm). The maximum root length (12.22) cm was recorded after KA9 + PDS1 + RS treatment, followed by KA-9 + RS (12.06 cm), PDS1 + RS (11.82 cm), BABA (11.32 cm), and mock (9.61 cm), and the minimum root length was recorded after treatment with RS (7.82 cm). Clustvis 2.0 online software was used to perform principal component analysis. Two main components PC1 and PC2 were observed to explain 99.80% of the variance. Growth parameter variables were substantially linked with PC1 and PC2 and were located in the same eclipse (excluding dry weight) at the 95% significance level. The results concluded that PDS1, KA9, and β-aminobutyric acid treatments, comprising eclipse I and eclipse II, showed significant positive responses to various growth parameters (Figure 12a).

KA9 + PDS1 + RS treatment resulted in the highest shoot dry weight of 1.23 g, followed by KA-9 + RS (1.18 g), PDS1 + RS (1.14 g), BABA (1.14 g), mock (0.98 g), and RS (0.68 g). The growth promotion efficiency (GPE) of shoots on a dry weight basis was calculated in comparison to the RS treatment, revealing the maximum value for KA9 + PDS1 + RS (80.87%) followed by KA-9 + RS (73.35%), BABA (69.11%), PDS1 + RS (67.64%), and mock (44.11%) (Table 5). The maximum root dry weight (0.32 g) was recorded after KA9 + PDS1 + RS and BABA treatments, followed by KA-9 + RS (0.31 g), PDS1 + RS (0.30 g), mock (0.28 g), and RS (0.18 g). The growth promotion efficiency of roots on a dry weight basis was calculated in comparison to the RS treatment, revealing the maximum value for KA9 + PDS1 + RS (77.76%), followed by BABA (77.00%), KA-9 + RS (72.22%), PDS1 + RS (66.67%), and mock (55.56%) (Figure 12b).

## 4. Discussion

The effects of volatile compounds (VOCs) produced by five rhizobacterial strains, PDS-1, UK-2, UK-4, BDS-1, and KA-9, on the growth and virulence features of the chili wilt pathogen *R. solanacearum* were investigated in this study. The VOCs produced by these isolates significantly inhibited the growth of *R. solanacearum* under in vitro conditions. The highest inhibition was recorded by strain PDS-1 (*P. fluorescens*) (72.24%) compared to the control. The results were concordant with the findings of Raza et al. [8], who reported that volatile organic compounds produced by *P*. *fluorescens* WR-1 restricted the growth and virulence traits of *R*. *solanacearum*. The effect of VOCs produced by different biocontrol strains on *R. solanacearum* has not been recorded; nevertheless, VOCs produced by the fungal pathogen *Aspergillus flavus* resulted in a fourfold decrease in *R. solanacearum* growth [34]. Other investigations found that *P. fluorescens* strains produced antimicrobial VOCs and suppressed the growth of the phytopathogen *Botrytis cinerea* [35]. *P. fluorescens* B-4117 produced VOCs that inhibited the growth of *Agrobacterium tumefaciens* and *Agrobacterium vitis* [36]. In this study, other strains belonging to the *Bacillus* genera showed significant inhibition activity against *R. solanacearum*, confirming the findings of Tahir et al. [10], who reported the effect of *Bacillus* volatile compounds against *R*. *solanacearum* in tobacco plant. Similar findings were also reported by Raza et al. [12], who showed the response of *R. solanacearum* to volatile organic compounds produced by biocontrol strain *B. amyloliquefaciens* SQR-9. The inhibitory activity of biocontrol agents in our study was due to presence of 2-ethyl-3,6-dimethyl pyrazine, 3-hydroxy-2-butanone (acetoin), benzothiazole, cycloheptasiloxane, and 2-methyl pyrazine compounds, in line with the results of Goa et al. [37], who identified pyrazine and benzothiazole as the significant inhibitory VOCs generated by *B*. *subtilis* CF-3.

### 4.1. Induction of Systemic Resistance by Rhizobacteria

This repeatedly showed that biocontrol agents such as *B. subtilis* KA9 and *P. fluorescens* PDS1 trigger a cascade of defense enzymes against *R. solanacearum*. To counter soil-borne diseases, rhizobacteria can induce systemic resistance in a range of plants, according to several studies. Plant growth-promoting bacteria (PGPB) use ISR to prime the entire plant body for disease resistance. Activation of latent defensive responses occurs not just at the induction site, but also systemically across plant tissues [38]. Plant defense enzymes such as polyphenol oxidase phenyl ammonia lyase and peroxidase can be activated by secondary metabolites generated by *Bacillus* species that induce systemic resistance [39]. PPO and PO are oxidative enzymes that can accelerate the synthesis of lignin and other oxidative phenols. The cell structure’s protective mechanism is changed to activate pathogen defense barriers [40].

According to the results of this study, in chili cv. Pusa Jwala, the combined treatment of *B. subtilis* KA9 and *P. fluorescens* PDS1 against *R. solanacearum* resulted in higher polyphenol oxidase, peroxidase, superoxide dismutase, and phenyl ammonia lyase activity, which increased up to 48 hapi, before gradually decreasing in all treatments. There was no increase in enzyme activity in the untreated control group. This result is supported by Jayapala et al. [39], who found that biopriming chili seeds with *Bacillus* sp. boosted defense-related enzymes such as PAL, peroxidase, and polyphenol oxidase at 48 hapi against *Colletotrichum capsici*. Chunyu et al. [41] found that treating tomato plants with *B. amyloliquefaciens* SQRT3 and *R. solanacearum* boosted PO and PPO activities; their findings were similar to the results of the current investigation. Pretreating tomato seedlings with *P. fluorescens* conferred systemic resistance against *Fusarium oxysporum* f.sp. *lycopersici*, according to Ramamoorthy et al. [42]. According to Kashyap et al. [33], biopriming chili seeds with rhizobacteria improved ISR against *R. solanacearum* by increasing peroxidase and phenyl ammonia lyase activities. According to Ho et al. [43], the jasmonic acid signaling pathway is important in the tomato’s defense mechanism against *R. solanacearum*. The findings of this study are backed up by observations of tomato plants treated with *B. subtilis* against the pathogen *Erwinia carotovora* subsp. *carotovora*, which boosted the activities of phenyl ammonia lyase and superoxide dismutase in seedlings [44]. Increased SOD causes more H_2_O_2_ to be deposited, which is required for plant disease resistance [45]. The majority of these data are in line with recent discoveries showing an increase in defense enzymes such PAL, PPO, POD, and SOD, which are involved in the prevention of plant disease [46]. Our findings matched those of Vanitha et al [47], who found that pretreatment with *P. fluorescens* enhanced the defense related enzyme activities like PAL, PO, Lipoxygenase PPO in tomato seedlings post inoculated with *R. solanacearum*.

### 4.2. Production of Volatile Compounds by Rhizobacteria

Microorganism-produced volatiles have been found to play an essential environmental role in the induction of systemic resistance to biotic elements, plant growth promotion, and bacterial pathogen suppression through beneficial interactions between PGPRs and plants [15]. Studies on bacterial volatile compounds (VOCs) and their effects on plant development and systemic resistance focused mostly on the interaction between PGPR-produced VOCs and plants [13]. The tripartite interaction involving microorganisms (PGPR strains *B. subtilis* KA9 and *P. fluorescens* PDS-1), a plant pathogenic bacterium (*R. solanacearum*), and the host chili plant was demonstrated in this study. Beneficial and pathogenic bacteria coexist in soil, and their efficacy as a PGPR or pathogen is influenced by the rhizosphere population, soil nutrition, and environmental variables. In soil, several relationships influence biological activity. In this work, the interaction effects of volatile compounds (VOCs) produced by both PGPR strains on the elicitation of defense reactions in the host chili were investigated. A significant difference was observed in all treatments of rhizobacteria with respect to defensive enzyme elicitation (PAL, PPO, PO, and SOD) when chili seedlings were exposed to volatile compounds produced by these rhizobacterial combinations in all experimental systems. During exposure to the pathogenic bacteria *R*. *solanacearum* VOCs, no huge variations were seen. In earlier studies, the inverted plate technique was utilized to determine whether VOCs can promote plant growth or induce systemic resistance. Generally, artificial growth medium is utilized in an inverted plate setup for plant growth studies; however, VOCs can only reach the leaves due to their solid or semisolid nature, leaving the roots unharmed. Another factor to consider is that, when pathogens are inoculated to test for induced systemic resistance, the pathogen grows on the growth medium surface and contaminates the inverted plate setup. To avoid this issue, we modified the method in this study by utilizing a half-inverted bottle system, in which soil mixed with vermiculite was employed as a plant development medium. VOCs could reach seedling roots through small pores in the soil–vermiculite combination in this transparent system. PGPR colonized soil organically, and the VOCs it produced could interact with the plant roots rather than leaves in natural settings. To investigate the growth-stimulating action of rhizobacteria VOCs in soil exposed to roots, the experiment was relocated from in vitro conditions to pots in a growth chamber with an RH of 80% and a temperature of 28 °C. The findings demonstrated that rhizobacterial VOCs encouraged development under soil conditions, and the results are supported by an earlier study by Park et al. [16]. Volatile organic compounds produced by microorganisms, caused many physiological changes related to defence enzymes, growth hormones, and photosynthesis [22]. Following exposure to rhizobacterial-VOCs, the results revealed a considerable increase in defense enzyme activity, implying that rhizobacterial VOCs can activate plant defensive mechanisms. Metabolites and defense-related enzymes enhance in concentration as a consequence of a buildup of resistance, such as PAL, according to molecular research. In addition to the generation of phenols, which operate in chemical defense, the enzyme PAL plays an important role in the regulation of lignin accumulation and the formation of defensive structures [48,49]. PPO catalyzes the oxygen-dependent oxidation of phenols, while PAL is implicated in the plant salicylic acid-mediated defense against pathogens. Both PPO and PAL are involved in the pathogenic microbe resistance process [50]. In response to pathogen attacks, plants exposed to VOCs increase their PPO and PAL levels. These findings corroborate recent research indicating an increase in the activity of PAL, PPO, and PO enzymes in *Bacillus*-treated tomato plants, which led to a decrease in wilt disease [51].

### 4.3. Expression of Rhizobacteria-Mediated Defense-Related Genes against R. solanacearum in Chili

Defense-related genes such as lipoxygenase (LOX), peroxidase (POD), phenyl ammonia lyase (PAL), superoxide dismutase (SOD), catalase (CAT), and defensin gene (DEF) were chosen to study their relative expression in the root and leaf tissue of chili induced by rhizobacteria and a chemical known to be directly associated with inducing systemic resistance through expression at both transcript and protein levels [52,53,54,55,56,57,58]. The WRKY (WRKYa, WRKY40) and HSF (HSFC) genes are transcription factors that act as activators or repressors of triggered immunity in the plant [59,60,61,62,63]. These genes are also involved in modulating gene expression during pathogen-associated molecular pattern-triggered immunity, as well as effector-triggered immunity. Furthermore, NPR1 (a nonexpressor of pathogenesis-related gene) contributes to the overall plant health when the immune response of the plant is increased [64,65,66]. Hence, qPCR was performed to study the relative expression of these genes in comparison with ubiquitin following different treatments with rhizobacteria and *R. solanacearum* in chili to understand the biology of systemic resistance induction.

#### 4.3.1. PAL gene

The relative expression of PAL gene in response to different treatments (RS (*R. solanacearum*), KA9 + RS (*B. subtilis* KA9+ *R. solanacearum*), PDS1 + RS (*P. fluorescens* PDS1 + *R. solanacearum*), KA9 + PDS1 + RS (*B. subtilis* KA9 + *P. fluorescens* PDS1 + *R. solanacearum*), BABA (chemical control), and mock inoculated treatments) on the Pusa Jwala cultivar of chili was studied. The BABA-treated leaf and root showed the highest relative expression of PAL gene at 48 h, which indicates that the priming of ISR maintains plant fitness when pathogens attack. In addition to chemical-induced priming of ISR, the priming effects can be elicited by rhizobacteria [67,68]. In this study, the relative expression of PAL gene was observed in response to different rhizobacteria treatments. PDS1 + RS and KA9 + RS treatments did not differ significantly, whereas the dual rhizobacterial treatment (KA9 + PDS1 + RS) was significantly better than the control and individual rhizobacterial treatments. The PAL gene was upregulated in all treatments compared to UBI3. These results agree with those reported by Chandrashekran and Chun [44], who tested the efficiency of *B. subtilis* CBR05 as a biocontrol agent for soft rot disease (*Erwinia carotovora* subsp*. carotovora*) in tomato and found that the PAL gene exhibited the most significant upregulation in the transcript profile. The general conclusion was that increases in PAL activity were caused by the increased expression of PAL gene. Previous studies [69,70,71,72] also reported that the PAL activity in hosts induced by microbial antagonists was linked to their biocontrol effects. When peach fruit was challenged with the pathogen *Colletotrichum acutatum*, Wang et al. [73] found that *B. cereus* AR156 treatment stimulated the expression of three defense-related genes, resulting in higher ISR and reduced anthracnose rot.

In this study, a similar pattern of relative expression of PAL gene was found in leaf samples, with BABA treatment showing the maximum fold expression, followed by the dual rhizobacterial inoculation (KA9 + PDS1 + RS). Upregulation of defensive gene PAL in leaves was found in all treatments compared to UBI3. The relative expression of PAL gene was greater in root tissue than in leaf tissue after all treatments. Kolahi and coworkers [74] reported a similar pattern of PAL gene expression in different tissues of sugarcane, with the lowest level recorded in leaf tissue compared to sheath and root tissues. This may be due to the lack of vascular bundles and secondary tissues in leaf, as the PAL gene is implicated in plant growth and development via the lignin synthesis pathway, related to changes in lignin content and composition, as well as cell-wall production [75,76].

#### 4.3.2. POD Gene

In the oxidation of phenolic compounds and protection against pathogens, POD is a key scavenger of H_2_O_2_ and works in tandem with PAL [77,78]. In this study, upregulation of defensive gene POD was found following all treatments compared to UBI3. The highest relative expression of POD gene was recorded after treatment with BABA, followed by KA9 + PDS1 + RS, which exhibited threefold increased expression of POD gene compared to UBI3. A similar result was reported by Malviya et al. [79], who showed that the activities of plant enzymes such as peroxidase and superoxide dismutase were significantly increased in plant roots after the inoculation of plant growth-promoting *Burkholderia anthina* MYSP113 in sugarcane crops. In this study, POD expression was higher in root tissue, which agrees with the studies of Bharti et al. [80], who reported that PGPR-treated wheat roots showed a 2–3-fold increase in the gene expression of antioxidants compared to control plants, with comparatively less POD gene expression observed in wheat leaf tissues. A similar observation was recorded by Prakash et al. [81], whereby *P. fluorescens* elicited defensive gene expression in tomato plant against *Alternaria* blight disease.

#### 4.3.3. SOD Gene

Superoxide dismutase (SOD) activity in plants is due to the expression of the corresponding SOD gene. SODs are important antioxidant enzymes that safeguard organisms against reactive oxygen species (ROS) produced under stress conditions [82]. In this study, a higher relative expression of SOD gene was recorded after dual rhizobacterial treatment compared to individual rhizobacterial treatments. The SOD gene was upregulated following all treatments compared to UBI3. Feng et al. [83] reported that *Sl-SOD1* gene sustained high expression in all tested tomato tissues during growth and development. They also recorded a higher expression in root tissue, in line with the results of this study. Corpas et al. [84] used qRT-PCR to examine the expression of SOD isozymes in various olive leaf cell types, including spongy mesophyll, palisade mesophyll, xylem, and phloem. The highest concentration of superoxide radicals was seen in vascular tissue, which had the lowest number of SOD transcripts. These findings revealed that, depending on the cell type of the olive leaf, each SOD isozyme had a varied gene expression, which could explain the lower SOD expression in leaf tissue seen in this study. Zhou et al. [82] also reported a similar pattern of SOD gene expression in different cucumber tissues under various biotic and abiotic stresses.

#### 4.3.4. WRKYa and WRKY40 Genes

WRKY is a conserved protein family implicated in a variety of biological processes in plants [85,86,87]. However, the mechanism underlying the functional diversity of WRKYs in chili has not been well elucidated. In this study, the relative expression of WRKYa gene exhibited a similar trend to that of other defensive genes, whereby dual rhizobacterial inoculation with *B. subtilis* KA9 and *P. fluorescens* PDS1 was found to be significantly better than other individual rhizobacterial treatments. Upregulation of defensive gene WRKYa was found after all treatments compared to UBI3 in root and leaf tissue. Upregulation of defensive gene WRKY40 was observed following BABA, PDS1 + KA9 + RS, and PDS1 + RS treatments, whereas downregulation was observed following KA9 + RS and RS treatments. This may be because WRKY transcription factors are interconnected with plant hormones and stress response pathways [88]. Nan and Gao [89] also analyzed gene expression using qRT-PCR and revealed that *WRKY* genes are involved in hormone and mechanic injury stresses. Nan et al. [90] characterized 39 orthologous gene pairs in rice WRKY (OsjWRKY) and performed qRT-PCR experiments to validate the tissue-specific and differential expression of OrWRKYs in response to abiotic stresses in leaves and roots; they found variable expression, in line with our results. Nan et al. [90] also studied the expression of 21 CaWRKY genes related to seven abiotic and biotic stimuli (heat shock, salt, drought, SA, *Phytophthora capsici*, ABA, and MeJA). Stress treatment increased the expression of several CaWRKY genes. According to Liu et al. [91], the WRKY40 transcription factor is known to positively influence the pepper plant’s defense response to *R. solanacearum*. For transcriptional control and production of the defense gene, WRKY40 binds directly to the W4-box element of the ChiIV3 promoter region. ChiIV3 is influenced by WRKY40 gene regulation in a variety of ways. This may be the reason for the lower disease incidence in our treatments where WRKY gene expression was high.

#### 4.3.5. Relative Expression of CAT2 Gene

Catalase is essential for the removal of H_2_O_2_ produced in the peroxisomes by photorespiration [92]. As a result of environmental stress, plant cells produce reactive oxygen species (ROS), which are free radicals that can damage membranes, oxidize proteins, and cause DNA damage [93,94]. Plants can be protected from biotic and abiotic stress conditions by altering the expression of ROS-scavenging enzymes such as peroxidase (POD), superoxide dismutase (SOD), and catalase (CAT) [95,96]. In this study, the highest relative expression of CAT2 gene was found in chili plants treated with BABA (3.75-fold). The relative expression observed after dual rhizobacterial treatment (KA9 + PDS1 + RS) was significantly better compared to individual rhizobacterial treatments. BABA was significantly different from rhizobacterial treatments. A different expression pattern in leaf and root tissue was recorded. A similar observation was also reported by Du et al. [97]. Greater expression of the CAT2 gene was detected in roots in this study, likely related to the sensitivity of catalase gene expression to the growth stage, light environment, and senescence [98], whereby different expression patterns may result from minor differences in developmental stages and growing circumstances.

Many studies have shown that stress activates catalase expression and activity in plants [99,100,101,102,103,104,105]. CAT1 exhibits very low levels of transcription and enzyme activity in normal conditions, but CAT2 may act as the main scavenger of H_2_O_2_ in response to stress, thereby playing an important role in adapting to environmental stresses. A greater incidence of wilting disease was recorded in chili plants with low CAT expression. A previous report indicated that deficiency of CAT2 reduced resistance in *Arabidopsis* plants [106].

#### 4.3.6. HFSC1 Gene

Chili is mainly grown by farmers in kharif season in India, with the summer temperature commonly exceeding 35 °C. Therefore, to determine the effect of heat stress on chili growth and development, we evaluated HFSC gene expression. The relative expression of HFSC1 gene was lower in roots than in leaf tissue. This may be because of the direct exposure of leaves to heat in glasshouse conditions [107,108,109]. The highest relative expression of HFSC1 in leaves was recorded after treatment with KA9 + PDS1 + RS compared to the UBI3. Increased expression was observed after KA9 + PDS1 + RS treatment compared to individual rhizobacterial treatments. Thus, dual inoculation with *B. subtilis* KA9 and *P*. *fluorescens* PDS1 may reduce the heat stress of chili plants and improve their growth, as indicated by the highest plant growth parameter. Ali et al. [110] corroborate this claim, demonstrating that PGPR inoculation reduced the negative effects of heat stress on plant growth and output. Mukhtar et al. [111] conducted a study to investigate the potential of heat-tolerant PGPR in mitigating heat stress effects in tomatoes, and they discovered that *B. cereus* was particularly successful in alleviating heat stress. For example, when subjected to diverse abiotic stimuli such as heat, salt, and heavy metals, Tang et al. [61] found that the expression patterns of 12 RsHSF genes changed significantly. In our results, we also noted different expression patterns of the HSFC gene, which could be due to temperature differences in the glasshouse during the summer.

#### 4.3.7. LOX2 Gene

Lipoxygenase (LOX) genes are abundant in plants and play an important role in biotic and abiotic stress resistance [112,113]. The LOX pathway’s products serve as growth regulators, antibacterial chemicals, flavors and odors, and signaling molecules, among other functions [114]. In this study, dual culture treatment KA9 + PDS1 + RS showed the highest relative expression of LOX gene in root tissue. In leaves, the highest relative expression of LOX2 gene was recorded after RS treatment, while the lowest expression was recorded after BABA treatment, compared to UBI3. A similar result was reported by Song et al. [112], who reported that *LOX* expression patterns differed significantly between wildtype peanut and cultivated peanut infected with *Aspergillus flavus*. Upregulation of defensive gene LOX2 in leaves was observed in all cases except after BABA treatment, compared to UBI3. A similar finding was also observed by Mariutto et al. [115] with CaLOX6 gene clusters, as SlLOXF is known to be involved in systemic resistance to *P*. *putida* BTP1. Downregulation of the LOX gene was recorded by Zhang et al. [116] in vegetative tissues and fruit, in line with this study. AdLox1 and AdLox5 expression increased dramatically as the fruit reached the climacteric stage and was upregulated by ethylene treatment, following a pattern similar to the LOX enzyme activity seen here. However, ethylene buildup was inversely correlated with the AdLox2, AdLox3, and AdLox4 transcript levels. This could explain why BABA treatment led to a downregulation of LOX gene, due to a fluctuation in the hormones required for BABA to confer resistance [117].

#### 4.3.8. Relative Expression of NPR1 Gene

NPR1 (nonexpresser of PR genes) is a master regulator of salicylic acid (SA) signaling, which is important for plant immunity. NPR1 interacts with transcription factors in the nucleus to boost defense responses by inducing the expression of PR (pathogenesis-related) genes [66]. In this study, the relative expression of NPR1 gene was highest after BABA treatment (3.13-fold). The downregulation of NPR1 gene in most cases indicates activation of a salicylic-acid-independent pathway, as less disease incidence was recorded, suggesting the induction of systemic resistance. As a master regulator of SA-mediated and JA-mediated plant immunity [118], NPR1 controls the expression of over 2000 genes [119,120]. Ramirez et al. [121] investigated the role of the *Arabidopsis* mutant OCP3 in SA- and JA-dependent induced defense and discovered that OCP3 plants were impaired by *P. fluorescens* WCS417-triggered induced systemic resistance (ISR) against both *P. syrinagae* DC3000 and *Hyaloperonospora arabidopsidis*. Thus, rhizobacteria may affect NPR1’s nucleocytosolic activity in the regulation of JA-dependent defensive responses. NPR1 facilitates the immunological transcriptome not only by activating SA-responsive genes but also by functioning as a corepressor of JA-responsive MYC2 [122].

## 5. Conclusions

Microbes can benefit agricultural systems by promoting plant growth and systemic resistance to diseases in plants without harming the environment. The plant growth-promoting efficiency of VOCs produced by *Pseudomonas fluorescens* PDS1 and *Bacillus subtilis* KA9 on chili against *Ralstonia solanacearum* was evaluated in a transparent closed assembly featuring a half-inverted plastic bottle setup, demonstrating the plant–microbial interactions via volatile compounds. The PDS1 and KA9 VOCs significantly increased defensive enzyme activity and overexpressed the antioxidant genes. The overall gene expression was higher in root tissue than in leaf tissue. Our findings shed light on the relationship among rhizobacteria, pathogens, and host plants, which promotes plant growth promotion, suppresses disease, induces systemic resistance, and triggers an antioxidant response in the leaves and roots of chili.

## Figures and Tables

**Table 1:** Inhibitory effect of volatile compounds produced by rhizobacterial isolates of chili against *Ralstonia solanacearum* under in vitro conditions.

Treatments	Colony Growth (mm)	Inhibition (%)
PDS1 + R	24.98	72.24
UK2 + R	32.08	64.35
UK4 + R	34.67	61.48
BDS1 + R	30.56	66.04
KA9 + R	28.45	68.38
Control	90.00	-
SEM (±)	1.41	
CD at 1%	4.58	

PDS1: *Pseudomonas fluorescens; UK2: Bacillus amyloliquefaciens*; UK4: *Bacillus cereus*; BDS1: *Bacillus subtilis*; KA9: *Bacillus subtilis*; R: *Ralstonia solanacearum*.

**Table 2:** Gas chromatography–mass spectrometry (GC–MS) profile of common volatile organic compounds (VOCs) produced in all the treatments during the study of rhizobacteria-mediated defensive enzymes in chili.

Volatile Compounds		Relative Peak Area (%)				
	Retention Time	T2 *(B*. *subtilis* KA9)	T3 (*P. fluorescens* PDS1)	T4 (*B*. *subtilis* KA9 + *R. solanacearum*)	T5 (*P. fluorescens* + *R. solanacearum*)	T7 (*R. solanacearum*)
Azulene	13.64	0.372 ± 0.22	0.255 ± 0.25	0.961 ± 0.32	0.119 ± 0.55	0.586 ± 0.22
Benzene, 1,2,3-trimethyl-	10.045	0.103 ± 0.20	0.730 ± 0.38	0.274 ± 0.02	0.419 ± 0.03	0.553 ± 0.55
Cycloheptasiloxane, tetradecamethyl-	18	0.152 ± 0.45	0.209 ± 0.22	0.170 ± 0.02	0.158 ± 0.52	0.111 ± 0.02
Cyclohexasiloxane, dodecamethyl-	15.605	0.117 ± 0.44	0.279 ± 0.42	0.157 ± 0.52	0.164 ± 0.32	0.107 ± 0.40
Cyclononasiloxane, octadecamethyl-	21.975	0.680 ± 0.33	0.196 ± 0.20	0.123 ± 0.05	0.177 ± 0.32	0.124 ± 0.22
Cyclooctasiloxane, hexadecamethyl-	20.135	0.408 ± 0.42	0.826 ± 0.50	0.542 ± 0.22	0.596 ± 0.02	0.463 ± 0.33
Cyclotrisiloxane, hexamethyl-	6.095	0.461 ± 0.42	0.617 ± 0.02	0.286 ± 0.33	0.386 ± 0.20	0.346 ± 0.22
Dodecane	13.74	0.271 ± 0.33	0.714 ± 0.22	0.252 ± 0.02	0.455 ± 0.02	0.229 ± 0.02
Ethylbenzene	7.05	0.330 ± 0.22	0.179 ± 0.33	0.782 ± 0.22	0.926 ± 0.44	0.338 ± 0.22
Nonane	7.96	0.354 ± 0.22	0.929 ± 0.55	0.159 ± 0.02	0.494 ± 0.55	0.199 ± 0.22
Tetradecane	16.715	0.412 ± 0.22	0.390 ± 0.55	0.154 ± 0.22	0.286 ± 0.02	0.134 ± 0.54
Toluene	4.675	0.584 ± 0.22	0.218 ± 0.44	0.290 ± 0.55	0.123 ± 0.22	0.202 ± 0.02
Undecane	12.035	0.133 ± 0.04	0.132 ± 0.33	0.384 ± 0.02	0.740 ± 0.02	0.358 ± 0.42

The compounds possibly generated by *R. solanacearum* treatments were identified by comparing their mass spectra to those in the NIST Mass Spectral Library (probability-based match >85%). Values are expressed as the means of the relative content of each compound (*n* = 3). The peak area of each compound was calculated as a percentage relative to the total peak area of all volatile organic compounds in a particular treatment. Several minor peaks are not included. RT, retention time. The values after ± indicate the standard deviations of three replicates.

**Table 3:** Gas chromatography–mass spectrometry (GC–MS) profile of major volatile organic compounds (VOCs) produced by different treatments of rhizobacteria.

Volatile Compounds	Retention Time	Relative Peak Area (%)			T5 (*P. fluorescens* + *R. solanacearum*)
		T2 *(B*. *subtilis* KA9)	T3 (*P. fluorescens* PDS1)	T4 (*B*. *subtilis* KA9 + *R. solanacearum*)	
2,6-Dimethylpyrazine	20.8	3.775061 ± 0.85	0.558445 ± 0.03	4.262294 ± 0.85	0.78169 ± 0.05
2-Methyl-1-butanol	14.3	1.400815 ± 0.25	0.235084 ± 0.05	1.876175 ± 0.55	0.262361 ± 0.33
4-Ethylbenzoic acid, 2-bromo-4-fluorophenyl	17.49	0.692943 ± 0.05	0.203268 ± 0.04	0.871781 ± 0.05	0.200583 ± 0.03
Anisole	12	0.612489 ± 0.05	0.621965 ± 0.05	0.100418 ± 0.02	0.543554 ± 0.03
Benzaldehyde, 3,5-dimethyl-	13.51	1.046637 ± 0.55	0.175852 ± 0.040	0.990622 ± 0.55	0.169998 ± 0.04
Benzothiazole	15.00	2.191521 ± 0.33	0.049163 ± 0.004	1.170364 ± 0.65	0.390761 ± 0.06
Butylthiophenol	11.9	0.601659 ± 0.005	0.264597 ± 0.005	0.55181 ± 0.005	0.2175 ± 0.005
Methylbutanal	8.2	0.407827 ± 0.02	0.111012 ± 0.02	0.534561 ± 0.02	0.126171 ± 0.05
Octanol	12.5	1.023067 ± 0.22	0.511885 ± 0.15	0.270572 ± 0.02	0.407792 ± 0.04
Oxime-, methoxy-phenyl-	8.195	0.325617 ± 0.33	0.079616 ± 0.020	0.543481 ± 0.05	0.072404 ± 0.005

The compounds possibly generated by rhizobacterial treatments were identified by comparing their mass spectra to those in the NIST Mass Spectral Library (probability-based match >85%). Values are expressed as the means of the relative content of each compound (*n* = 3). The peak area of each compound was calculated as a percentage relative to the total peak area of all volatile organic compounds in a particular treatment. Several minor peaks are not included. RT, retention time. The values after ± indicate the standard deviations of three replicates.

**Table 4:** Gas chromatography–mass spectrometry (GC–MS) profile of common volatile organic compounds (VOCs) produced by the treatment (T7) with *R. solanacearum*.

Volatile Compounds	Retention Time	Relative Peak Area (%)
1,2-Benzenedicarboxylic acid, bis (2-methylpropyl) ester	8.04	0.38 ± 0.44
2,4-Dimethyl-1-heptene	10.64	0.18 ± 0.03
2-Nonenal, (*E*)-	12.04	0.12 ± 0.43
3-Heptene, 2,2,4,6,6-pentamethyl-	16	0.17 ± 0.03
5-Hepten-2-one, 6-methyl-	14.64	0.80 ± 0.22
Benzene, (1,3,3-trimethylnonyl)-	18.90	0.08 ± 0.05
Benzene, (1-methylethyl)-	20.11	0.61 ± 0.33
Benzene, 1,1’-(1-methylethylidene) bis [4-methyl-	18.05	0.71 ± 0.05
Benzene, 1,3-dichloro-	18.74	0.90 ± 0.22
Benzene, 1-ethyl-3-methyl-	9.05	0.32 ± 0.02
Benzene, propyl-	6.96	0.42 ± 0.22
Butyric acid, 2-phenyl-, dec-2-yl ester	17.15	0.55 ± 0.22
Decanal	8.65	0.33 ± 0.03
Decane, 3,7-dimethyl-	10.03	0.30 ± 0.03
Decane, 4-methyl-	10.08	0.28 ± 0.05
Dodecane, 2,6,11-trimethyl-	12.18	0.82 ± 0.05
Eucalyptol	6.04	0.67 ± 0.41
Heptadecane	2.05	0.90 ± 0.04
Heptanal	2.90	0.08 ± 0.03
Heptane, 2,3-dimethyl-	2.80	0.61 ± 0.52
Hexanal	6.09	0.79 ± 0.03
Indane	12.05	0.20 ± 0.02
Isopropyl myristate	14.08	0.92 ± 0.02
Nonanal	10.30	0.12 ± 0.04
Nonane, 5-butyl-	12.08	0.98 ± 0.02
Octanal	6.04	0.22 ± 0.03
Octane	8.08	0.77 ± 0.05
Octane, 2,3,6,7-tetramethyl-	10.14	0.90 ± 0.05

The compounds possibly generated by *R. solanacearum* treatment were identified by comparing their mass spectra to those in the NIST Mass Spectral Library (probability-based match >85%). Values are expressed as the means of the relative content of each compound (*n* = 3). The peak area of each compound was calculated as a percentage relative to the total peak area of all volatile organic compounds in a particular treatment. Several minor peaks are not included. RT, retention time. The values after ± indicate the standard deviations of three replicates.

**Table 5:** Evaluation of potential of antagonistic rhizobacteria in terms of growth promotion of chili 40 days after inoculation in glasshouse conditions.

Strains Treatment/Growth Trait	Wilt Disease Incidence (%)	Biocontrol Efficacy (%)	Shoot Length (cm)	Shoot Fresh Weight (g)	Shoot Dry Weight (g)	GPE (%)	Root Length (cm)	Root Fresh Weight (g)	Root Dry Weight (g)	GPE (%)
**RS**	78 ^a^	-	10.3 ^f^	7.2 ^f^	0.68 ^e^	-	7.82 ^f^	0.88 ^f^	0.18 ^e^	-
**MOCK**	0 ^e^	-	16.6 ^e^	8.5 ^e^	0.98 ^d^	44.11	9.61 ^e^	1.76 ^e^	0.28 ^d^	55.56
**KA9 + RS**	26.50 ^b^	66.02 ^b^	19.8 ^c^	9.8 ^c^	1.18 ^b^	73.35	12.06 ^b^	1.87 ^c^	0.31 ^b^	72.22
**PDS1 + RS**	26.80 ^b^	65.64 ^c^	18.6 ^d^	8.7 ^d^	1.14 ^c^	67.64	11.82 ^c^	1.79 ^d^	0.30 ^c^	66.67
**KA9 + PDS1 + RS**	22.40 ^c^	71.28 ^a^	21.7 ^a^	11.5 ^a^	1.23 ^a^	80.88	12.22 ^a^	1.89 ^b^	0.32 ^a^	77.78
**BABA**	21.80 ^d^	72.05 ^a^	20.8 ^b^	10.9 ^b^	1.14 ^c^	69.11	11.32 ^d^	1.96 ^a^	0.32 ^a^	77.00

Values given in column are the average of three replications. Values with different alphabetical (a–f) superscripts within a column are significantly different as determined by LSD test (α = 0.05).

## Data Availability

Data is contained within the article or Supplementary Material.

## Supplementary Materials

The following are available online at https://www.mdpi.com/article/10.3390/antiox11020404/s1, Supplementary Figure S1; Supplementary Figure S2; Supplementary Figure S3a–e; Supplementary Table S1; Supplementary Table S2; Supplementary Table S3; Supplementary Table S4; Supplementary Table S5; Supplementary Table S6; Supplementary Table S7; Supplementary Table S8; Supplementary Table S9; Supplementary Table S10; Supplementary Table S11; Supplementary Table S12; Supplementary M and M.
