# Supplementary material for: Unraveling Microbial Volatile Elicitors Using a Transparent Methodology for Induction of Systemic Resistance and Regulation of Antioxidant Genes at Expression Levels in Chili against Bacterial Wilt Disease
Source: Antioxidants (Basel). 2022 Feb 16;11(2):404. doi: 10.3390/antiox11020404 (PMC8869530; doi:10.3390/antiox11020404)
Supplement: Supplementary file 1 [file antioxidants-11-00404-s001.zip › suplementary M and M.pdf]

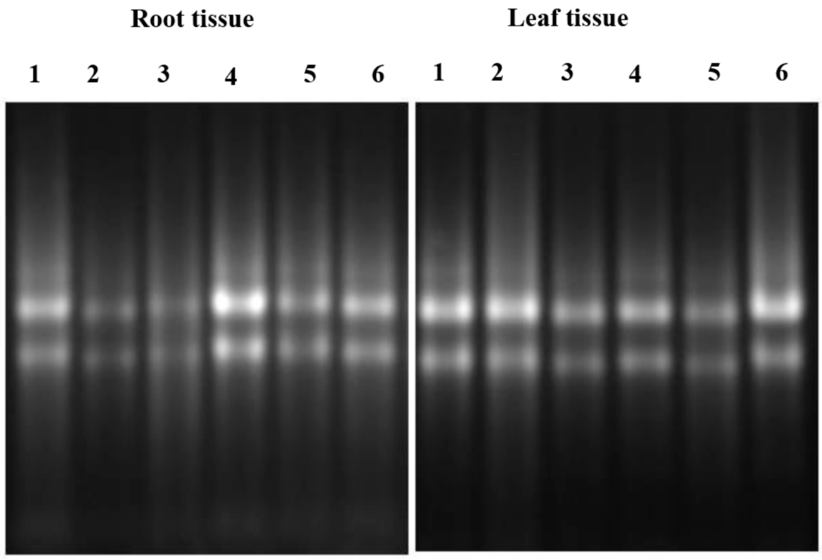

Figure Confirmation of RNA integrity after the extraction from the chilli leaf and root tissue by electrophoretic separation on 1.2% agarose gel. Lane 1-6 RNA samples from different treatments *viz*. mock, RS (*R. solanacearum*), KA9 + RS (*Bacillus subtilis* + *R. solanacearum*), PDS1 + RS (*Pseudomonas fluorescens* + *R. solanacearum*), KA9+PDS1+RS (*Bacillus subtilis* + *Pseudomonas fluorescens* + *R. solanacearum*) and BABA (chemical control).

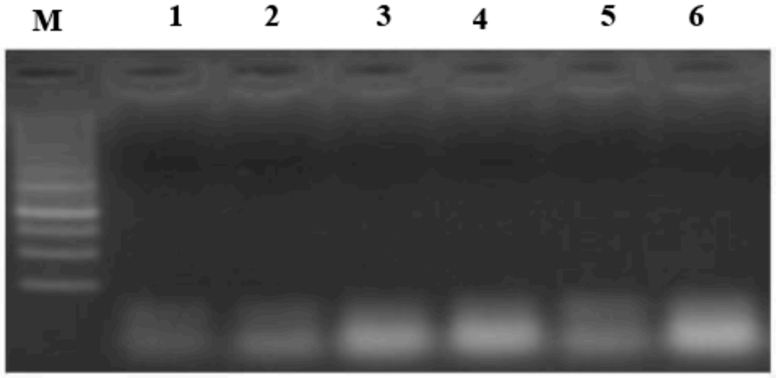

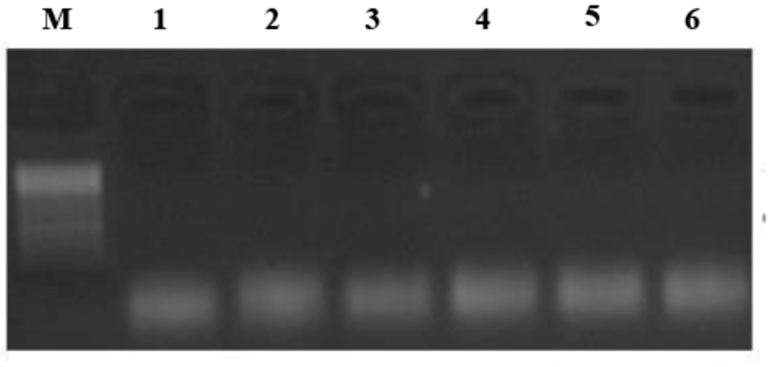

Figure RNA-PCR to check DNA contamination, isolated RNA from root and leaf tissue of chilli has been given treatment with DNase to remove the residual DNA, checked for the presence of DNA contamination using UBI3 gene-specific primers. Do not observe the presence of amplicons in all the samples analyzed. Lane M- 1 Kb marker, lanes 1-6 RNA samples from different treatments *viz*. mock, RS (*R. solanacearum*), KA9 + RS (*B. subtilis* + *R. solanacearum*), PDS1 + RS (*P. fluorescens* + *R. solanacearum*), KA9+PDS1+RS (*B. subtilis* + *P. fluoresecens* + *R. solanacearum*) and BABA (chemical control).

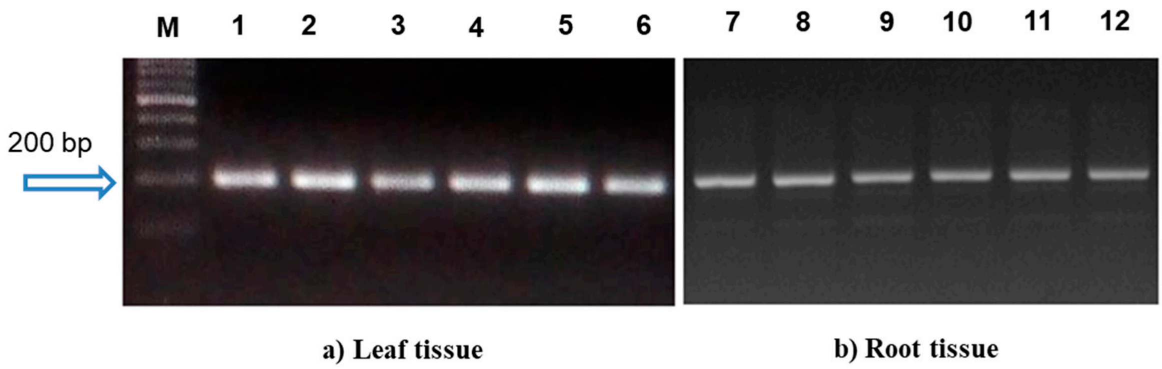

Figure 15. Ubi3 RT-PCR to check cDNA quality, Reverse transcriptase-PCR successfully amplified at 200 bp from the cDNA synthesized from the RNA samples, thus confirming the functionality of the cDNA samples. Lane M- 1Kb marker, Lanes 1-6 cDNA samples synthesized from the RNA extracted from the different treatments of chilli leaf (Lines 1-6) and root samples (lines 7-12).

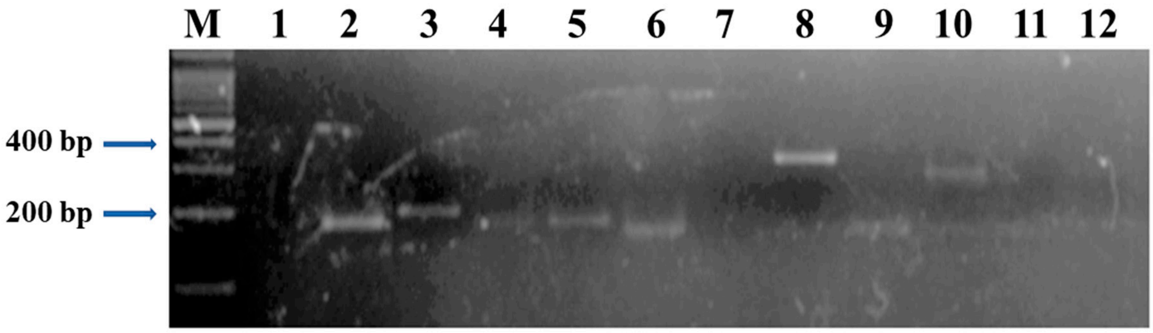

Figure. Gene specific primers amplification check on cDNA, confirmation of the presence of transcripts for PAL, POD, SOD, WRKYa, PAL1, DEF-1, CAT-2, WRKY40, HSFC1, LOX2,

NPR1 and Ubiquitin 3 in pooled cDNA samples of leaf and root tissues. Specific size of amplicon for each of the transcript's reverse transcriptase-PCR successfully amplified >150 bp to <300 bp gene product from the cDNA synthesized from the RNA samples, thus confirming the functionality of the cDNA samples. Lane M- 100 bp marker, Lanes 1-12 different gene specific primers amplification check on cDNA.
