# Supplementary material for: Unraveling Microbial Volatile Elicitors Using a Transparent Methodology for Induction of Systemic Resistance and Regulation of Antioxidant Genes at Expression Levels in Chili against Bacterial Wilt Disease
Source: Antioxidants (Basel). 2022 Feb 16;11(2):404. doi: 10.3390/antiox11020404 (PMC8869530; doi:10.3390/antiox11020404)
Supplement: Supplementary file 1 [file antioxidants-11-00404-s001.zip › suplementary Table S1.pdf]

Table 4.2.9. Supplementary Table S1 Effect of volatile compound produce by biocontrol agents on induction of Peroxidase activities in chilli leaves against *R. solanacearum* in glasshouse conditions.

**Treatments**	**Peroxidase activity (ΔOD/min/mg protein)**					
	**0hapi**	**24hapi**	**48hapi**	**72hapi**	**96hapi**	**Mean value**
T1 (Control)	0.012 ± 0.001^{bc}	0.017 ± 0.002^{d}	0.021 ± 0.001^{f}	0.019 ± 0.002^{d}	0.017 ± 0.002^{d}	0.017
T2 (*B. subtilis* KA9)	0.012 ± 0.001^{bc}	0.029 ± 0.002^{c}	0.052 ± 0.002^{e}	0.048 ± 0.002^{c}	0.038 ± 0.003^{c}	0.035
T3 (*P. fluorescens* PDS1)	0.015 ± 0.001^{a}	0.027 ± 0.003^{c}	0.064 ± 0.003^{cd}	0.061 ± 0.002^{ab}	0.042 ± 0.002^{bc}	0.041
T4 (*B. subtilis* KA9 + RS*)*	0.014 ± 0.001^{ab}	0.046 ± 0.003^{b}	0.069 ± 0.003^{c}	0.056 ± 0.002^{b}	0.051 ± 0.002^{a}	0.047
T5 (*P. fluorescens* + RS*)*	0.013 ± 0.001^{ab}	0.049 ± 0.002^{b}	0.080 ± 0.002^{b}	0.058 ± 0.002^{ab}	0.048 ± 0.002^{ab}	0.049
T6(RS+ KA9+ PDS1)	0.014 ± 0.001^{ab}	0.056 ± 0.003^{a}	0.090 ± 0.002^{a}	0.064 ± 0.002^{a}	0.051 ± 0.002^{a}	0.055
T7 (RS)	0.013 ± 0.001^{ab}	0.029 ± 0.002^{c}	0.059 ± 0.002^{d}	0.043 ± 0.002^{c}	0.045 ± 0.002^{b}	0.037
Mean value	0.013	0.036	0.062	0.049	0.041	-
C.D.	0.002	0.007	0.007	0.006	0.006	-
SE(m)	0.001	0.002	0.002	0.002	0.002	-
SE(d)	0.001	0.003	0.003	0.003	0.003	-
C.V.	9.627	14.266	8.371	8.929	11.189	-
