# Supplementary material for: Unraveling Microbial Volatile Elicitors Using a Transparent Methodology for Induction of Systemic Resistance and Regulation of Antioxidant Genes at Expression Levels in Chili against Bacterial Wilt Disease
Source: Antioxidants (Basel). 2022 Feb 16;11(2):404. doi: 10.3390/antiox11020404 (PMC8869530; doi:10.3390/antiox11020404)
Supplement: Supplementary file 1 [file antioxidants-11-00404-s001.zip › suplementry Table S3.pdf]

Table 4.2.11. Supplementary Table S3 Effect of volatile compound produce by biocontrol agents on induction of Polyphenyloxidase activities in chilli leaves against *R. solanacearum* in glasshouse condition

**Treatments**	**Polyphenol oxidase activity (ΔOD/min/mg protein)**					
	**0hapi**	**24hapi**	**48hapi**	**72hapi**	**96hapi**	**Mean value**
T1 (Control)	0.012±0.001^{b}	0.016±0.001^{f}	0.022±0.002^{f}	0.019±0.001^{d}	0.018±0.002^{d}	0.017
T2 (*B. subtilis* KA9)	0.012±0.001^{b}	0.026±0.001^{e}	0.043±0.001^{e}	0.051±0.004^{bc}	0.032±0.001^{c}	0.032
T3 (*P. fluorescens* PDS1)	0.015±0.001^{a}	0.032±0.001^{d}	0.060±0.001^{d}	0.051±0.001^{bc}	0.037±0.003^{bc}	0.039
T4 (*B. subtilis* KA9 + RS*)*	0.012±0.001^{b}	0.043±0.001^{c}	0.064±0.001^{c}	0.060±0.003^{ab}	0.041±0.003^{ab}	0.044
T5 (*P. fluorescens* + RS*)*	0.012±0.001^{b}	0.047±0.001^{b}	0.074±0.001^{b}	0.054±0.004^{bc}	0.041±0.003^{ab}	0.045
T6(RS+ KA9+ PDS1)	0.013±0.001^{b}	0.055±0.001^{a}	0.082±0.001^{a}	0.064±0.003^{a}	0.048±0.003^{a}	0.052
T7 (RS)	0.012±0.001^{b}	0.027±0.001^{e}	0.045±0.003^{e}	0.049±0.002^{c}	0.044±0.002^{ab}	0.035
Mean value	0.012	0.035	0.055	0.049	0.037	-
C.D.	0.002	0.002	0.004	0.008	0.008	-
SE(m)	0.001	0.001	0.001	0.003	0.003	-
SE(d)	0.001	0.001	0.002	0.004	0.004	-
C.V.	9.712	4.407	5.664	12.544	15.400	-
