# Supplementary material for: Unraveling Microbial Volatile Elicitors Using a Transparent Methodology for Induction of Systemic Resistance and Regulation of Antioxidant Genes at Expression Levels in Chili against Bacterial Wilt Disease
Source: Antioxidants (Basel). 2022 Feb 16;11(2):404. doi: 10.3390/antiox11020404 (PMC8869530; doi:10.3390/antiox11020404)
Supplement: Supplementary file 1 [file antioxidants-11-00404-s001.zip › supplemantry Table S2.pdf]

Supplementary Table S2 Effect of volatile compound produce by biocontrol agents on induction of peroxidase (PO) activities in chilli Roots against *R. solanacearum* in glasshouse condition

Treatments	Peroxidase activity (ΔOD/min/mg protein)					
	**0 hapi**	**24hapi**	**48hapi**	**72hapi**	**96hapi**	**Mean value**
T1 (Control)	0.0019±0.030d	0.0031±0.029f	0.0028±0.021f	0.0027±0.021d	0.0028±0.020c	0.026
T2 (*B. subtilis* KA9)	0.0022±0.024c	0.0045±0.032e	0.0051±0.014e	0.0042±0.027b	0.0028±0.026c	0.037
T3 (*P. fluorescens* PDS1)	0.0028±0.027b	0.0050±0.022d	0.0060±0.014d	0.0050±0.031a	0.0038±0.019a	0.045
T4 (*B. subtilis* KA9 + RS*)*	0.0034±0.023a	0.0060±0.027b	0.0074±0.022c	0.0043±0.039b	0.0033±0.024b	0.048
T5 (*P. fluorescens* + RS)	0.0031±0.024a	0.0058±0.021c	0.0082±0.020b	0.0033±0.02c	0.0028±0.022c	0.046
T6(RS+KA9+PDS1)	0.0034±0.025a	0.0073±0.021a	0.0092±0.02a	0.0033±0.024c	0.0025±0.022d	0.051
T7 (RS)	0.0024±0.008c	0.0052±0.027d	0.0077±0.020c	0.0036±0.021c	0.0028±0.030c	0.043
Mean vlue	0.027	0.052	0.066	0.037	0.029	-
