# Supplementary material for: Unraveling Microbial Volatile Elicitors Using a Transparent Methodology for Induction of Systemic Resistance and Regulation of Antioxidant Genes at Expression Levels in Chili against Bacterial Wilt Disease
Source: Antioxidants (Basel). 2022 Feb 16;11(2):404. doi: 10.3390/antiox11020404 (PMC8869530; doi:10.3390/antiox11020404)
Supplement: Supplementary file 1 [file antioxidants-11-00404-s001.zip › supplementary Figure S3.pdf]

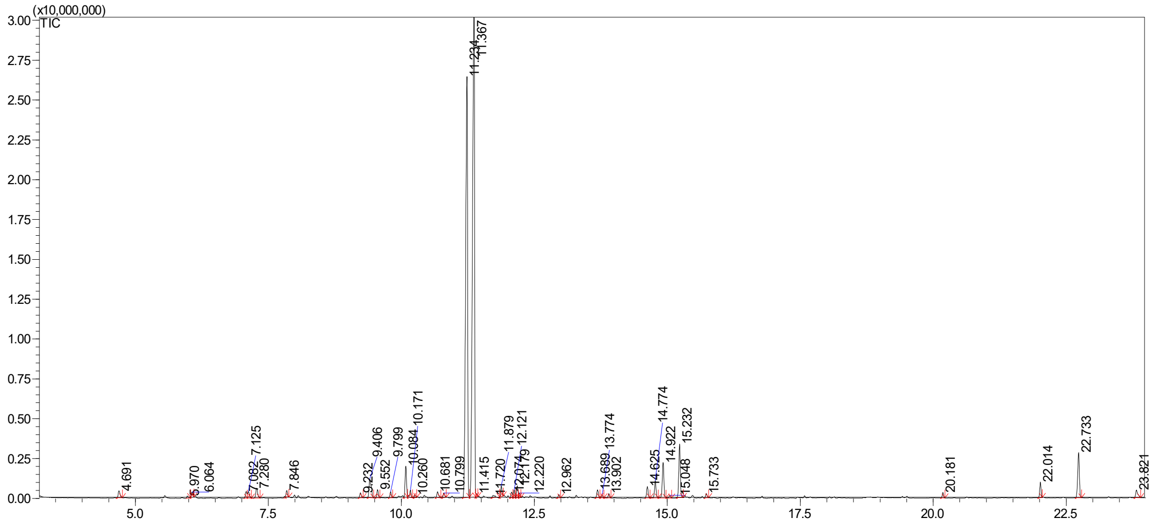

Supplementary Figure S3.(a) Chromatographic separation of VOCs of rhizobacteria treatment T2 (*B. subtilis* KA9).

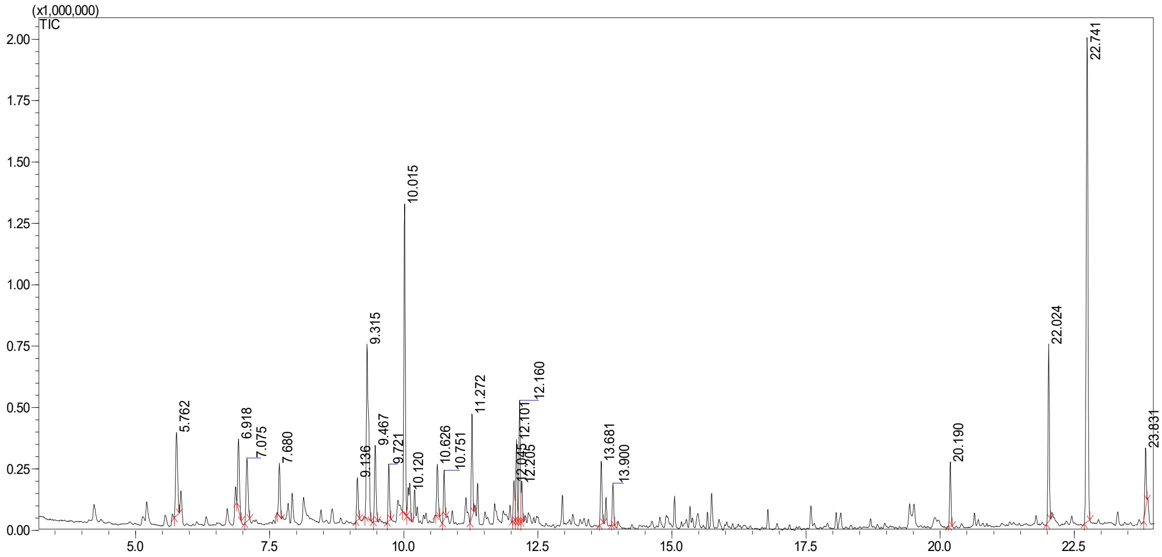

Supplementary Figure S3 (b)**.** Chromatographic separation of VOCs of rhizobacteria treatment T5 (*P. fluorescens* + *R. solanacearum*).

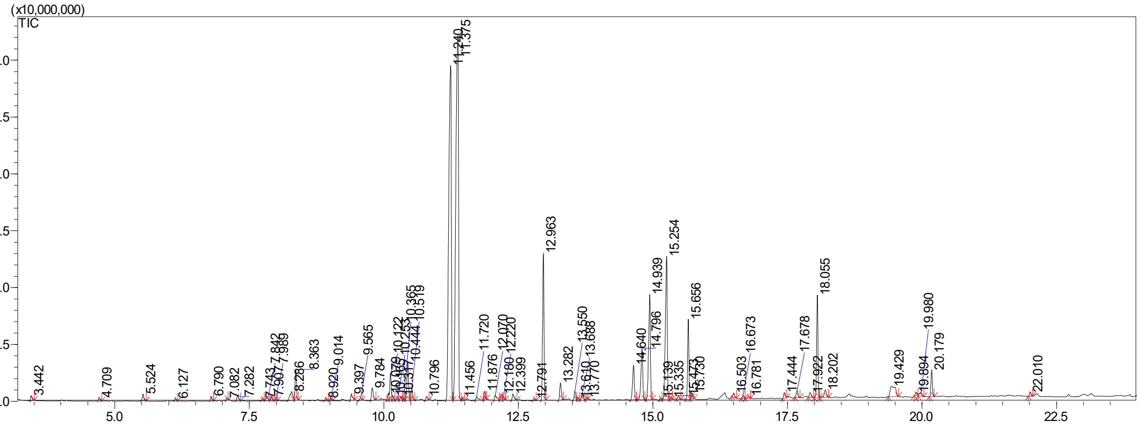

Supplementary Figure S3 (c). Chromatographic separation of VOCs of rhizobacteria treatment T7 (*Ralstonia solanacearum*).

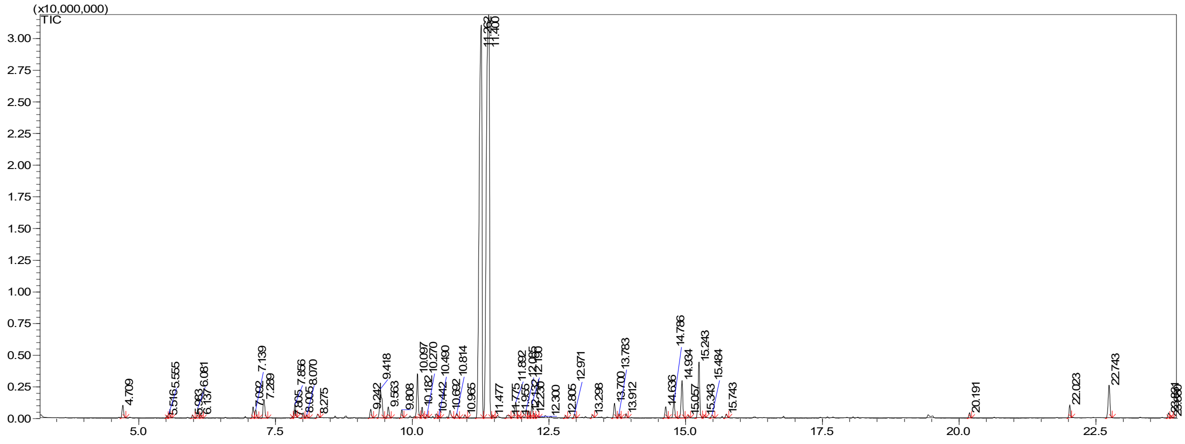

Supplementary Figure S3 (d). Chromatographic separation of VOCs of rhizobacteria treatment T3 (*P. fluorescens PDS1)*.

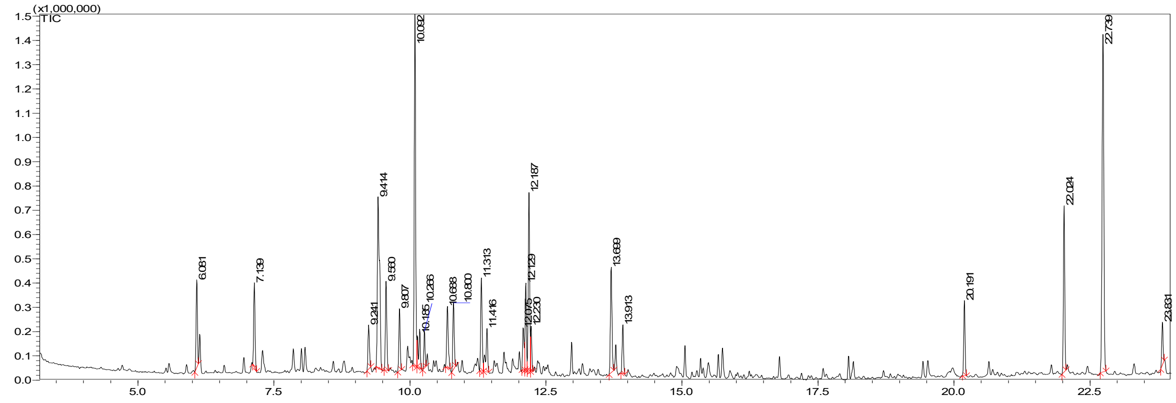

Supplementary Figure S3 (e). Chromatographic separation of VOCs of rhizobacteria treatment T4 (*B. subtilis* KA9+ *R. solanacearum*)
