# Supplementary material for: Unraveling Microbial Volatile Elicitors Using a Transparent Methodology for Induction of Systemic Resistance and Regulation of Antioxidant Genes at Expression Levels in Chili against Bacterial Wilt Disease
Source: Antioxidants (Basel). 2022 Feb 16;11(2):404. doi: 10.3390/antiox11020404 (PMC8869530; doi:10.3390/antiox11020404)
Supplement: Supplementary file 1 [file antioxidants-11-00404-s001.zip › supplementary Table S10.pdf]

Table S10 4.3.3. Relative expression levels (log fold change) of defense-related genes by strain PDS1 and KA9 different treatments in chilli leaf tissue following rhizobacteria inoculation and pathogen challenge after 48 hours.

**Defense-related genes**	**Relative expression levels (log fold change) Minus ddCT**					
	**Mock**	**RS**	**KA-9+ RS**	**PDS-1 + RS**	**KA-9+PDS-1 + RS**	**CC**
PAL	0.5^{b}	2.04^{a}	2.75^{a}	2.59^{a}	3.25^{a}	4.28^{a}
POD	0.3^{d}	1.96^{c}	1.99^{b}	2.1^{b}	2.39^{c}	3.15^{c}
SOD	1^{a}	2.03^{a}	1^{h}	1.62^{d}	2.7^{b}	3.6^{b}
WRKYa	0.2^{e}	0.32^{h}	0.8^{k}	0.82^{i}	1.2^{g}	1.9^{h}
PAL1	-0.5^{g}	0.86^{f}	1.5^{d}	1.9^{c}	1.57^{h}	2^{g}
DEF1	-1.5^{h}	1.2^{d}	1.7^{c}	0.9^{h}	1.61^{g}	3^{d}
CAT2	-0.9^{i}	0.6^{g}	1.2^{f}	1.34^{e}	1.91^{d}	2.7^{e}
WRKY40	0.4^{c}	-0.65^{i}	1.3^{e}	1.4^{f}	0.9^{i}	1.5^{i}
HSFC1	0.2^{f}	0.9^{e}	0.9^{i}	0.8^{j}	1.5^{f}	0.87^{j}
LOX2	1^{a}	1.98^{b}	1.07^{g}	1.04^{g}	1.84^{e}	-0.66^{k}
NPR1	0.2^{f}	-0.93^{k}	0.67^{j}	-0.22^{k}	0.74^{j}	2.15^{f}
