# Supplementary material for: Unraveling Microbial Volatile Elicitors Using a Transparent Methodology for Induction of Systemic Resistance and Regulation of Antioxidant Genes at Expression Levels in Chili against Bacterial Wilt Disease
Source: Antioxidants (Basel). 2022 Feb 16;11(2):404. doi: 10.3390/antiox11020404 (PMC8869530; doi:10.3390/antiox11020404)
Supplement: Supplementary file 1 [file antioxidants-11-00404-s001.zip › supplementary Table S11.pdf]

Table S11. Gas chromatography-mass spectrometry (GC-MS) profile of volatile organic compounds (VOCs) produced by rhizobacteria against *R. solanacearum* under *in vitro* conditions.

**Compounds**	**Retention Time**	**Peak Area (%)**	
		***Bacillus subtilis* KA9**	***Pseudomonas fluorescens* PDS1**
2-ethyl 3, 6-dimethyl pyrazine	13.44	22.59 ±0.04	28.07±0.33
3-hydroxy-2-butanone (acetoin)	10.045	13.68±0.33	10.02±0.94
Benzaldehyde	18	0.32±0.44	0.43±0.04
Cycloheptasiloxane	17.605	0.73±0.05	-
2-methyl pyrazine	22.975	15.95±0.55	24.40±0.22
1-Undecene	11.165	-	7.10±0.88
Diethylphthalate	6.095	7.75±0.33	7.10±0.04
Cyclohexanone	12.722	0.22±0.02	0.20±0.33
(1,2,α) pyrazine-1,4-dione	21.052	3.96±0.22	-
4-Hydroxy-5-methyl-2-hexanone	07.966	0.43±0.38	0.87±0.07
2,4-di-tert-Butylphenol	10.709	0.47±0.44	0.46±0.44
Hexadecane	4.675	0.98±0.05	0.23±0.02
Hexahydropyrrolo (1,2,α) pyrazine-1,4-dione	18.025	4.78±0.54	3.23±0.02
Isoamyl alcohol	2.084	10.07±0.04	20.09±0.02
Disulphide, dimethyl	7.0123	2.87±0.08	6.34±0.14

The compounds possibly generated by rhizobacteria treatments were identified by comparing their mass spectra to those in the NIST Mass Spectral Library (probability-based match >85%). Values are expressed as means of the relative content of each compound (n = 3). The peak area of each compound was calculated in percentage relative to the total peak area of all volatile organic compounds in a particular treatment. Several minor air-

peaks were not included. RT, retention time. The values after ± indicate the standard deviations of three replicates.
