# Supplementary material for: Unraveling Microbial Volatile Elicitors Using a Transparent Methodology for Induction of Systemic Resistance and Regulation of Antioxidant Genes at Expression Levels in Chili against Bacterial Wilt Disease
Source: Antioxidants (Basel). 2022 Feb 16;11(2):404. doi: 10.3390/antiox11020404 (PMC8869530; doi:10.3390/antiox11020404)
Supplement: Supplementary file 1 [file antioxidants-11-00404-s001.zip › Supplementary Table S12.pdf]

Table S12. Population of *R. solanacearum* in the rhizosphere soil of chilli plant under different treatment

**Treatments/hours**	**Population of *R. solanacearum* (log CFU/g root)**				
	**0hapi**	**24hapi**	**48hapi**	**72hapi**	**96hapi**
Control	0^{d}	0^{e}	0^{e}	0^{e}	0^{e}
KA9+RS	2 ± 0.04^{c}	2.5 ± 0.06^{c}	1.75 ± 0.02^{c}	1.59 ± 0.01^{b}	1.52 ± 0.02^{c}
PSD1+RS	2.5 ± 0.06^{a}	2.9 ± 0.13^{b}	2.2 ± 0.04^{b}	1.9 ± 0.03^{c}	1.9 ± 0.01^{b}
RS + KA9+PDS1	2.2 ± 0.03^{b}	2 ± 0.06^{d}	1.2 ± 0.09^{d}	1.2 ± 0.08^{d}	1 ± 0.06^{d}
RS	2 ± 0.29^{c}	3.1 ± 0.09a	5.9 ± 0.06^{a}	5.4 ± 0.05^{a}	4 ± 0.16^{a}

Means followed by the same letter within a column are not significantly different as determined by LSD test (α = 0.05). Data present means of the experiment within three replications each.
