# Supplementary material for: Unraveling Microbial Volatile Elicitors Using a Transparent Methodology for Induction of Systemic Resistance and Regulation of Antioxidant Genes at Expression Levels in Chili against Bacterial Wilt Disease
Source: Antioxidants (Basel). 2022 Feb 16;11(2):404. doi: 10.3390/antiox11020404 (PMC8869530; doi:10.3390/antiox11020404)
Supplement: Supplementary file 1 [file antioxidants-11-00404-s001.zip › supplementary Table S4.pdf]

Table 4.2.12. Supplementary Table S4 Effect of volatile compound produce by biocontrol agents on induction of Polyphenyloxidase activities in chilli roots against *R. solanacearum* in glasshouse conditions.

**Treatments**	**Polyphenol oxidase activity (ΔOD/min/mg protein)**					
	**0 hapi**	**24 hapi**	**48hapi**	**72hapi**	**96hapi**	**Mean value**
T1 (Control)	0.0019±0.017^{b}	0.0020±0.025^{e}	0.0019±0.017^{d}	0.0023±0.021^{e}	0.0025±0.026^{e}	0.0021
T2 (*B. subtilis* KA9)	0.0021±0.024^{a}	0.0033±0.022^{d}	0.0027±0.031^{c}	0.0052±0.032^{c}	0.0042±0.028^{c}	0.0035
T3 (*P. fluorescens* PDS1)	0.0023±0.025^{a}	0.0042±0.025^{c}	0.0032±0.026^{c}	0.0064±0.033^{b}	0.0044±0.029^{b}	0.0041
T4 (*B. subtilis* KA9 + RS*)*	0.0022±0.026^{a}	0.0054±0.028^{b}	0.0038±0.027^{b}	0.0058±0.033^{b}	0.0046±0.028^{a}	0.0043
T5 (*P. fluorescens* + RS*)*	0.0019±0.029^{b}	0.0058±0.032^{b}	0.0038±0.031^{b}	0.0063±0.034^{b}	0.0046±0.026^{a}	0.0044
T6(RS+ KA9+ PDS1)	0.0020±0.025^{b}	0.0067±0.030^{a}	0.0043±0.028^{a}	0.0075±0.034^{a}	0.0048±0.031^{a}	0.0050
T7 (RS)	0.0023±0.025^{a}	0.0035±0.026^{d}	0.0029±0.026^{c}	0.0042±0.020^{d}	0.0033±0.022^{d}	0.0032
Mean value	0.0021	0.0044	0.0032	0.0053	0.0040	-
