# Supplementary material for: Unraveling Microbial Volatile Elicitors Using a Transparent Methodology for Induction of Systemic Resistance and Regulation of Antioxidant Genes at Expression Levels in Chili against Bacterial Wilt Disease
Source: Antioxidants (Basel). 2022 Feb 16;11(2):404. doi: 10.3390/antiox11020404 (PMC8869530; doi:10.3390/antiox11020404)
Supplement: Supplementary file 1 [file antioxidants-11-00404-s001.zip › supplementary Table S5.pdf]

Table.4.2.13. **Supplementary Table S5** Effect of volatile compound produce by biocontrol agents s on induction of Superoxide dismutase activities in chilli leaves against *R. solanacearum* in glasshouse conditions.

**Treatments**	**Superoxide dismutase activity (ΔOD/min/mg protein)**					
	**0hapi**	**24hapi**	**48hapi**	**72hapi**	**96hapi**	**Mean value**
**T1 (Control)**	0.022±0.001^{ab}	0.027±0.003^{d}	0.030±0.003^{d}	0.027±0.003^{c}	0.021±0.002^{c}	0.025
**T2 (*B. subtilis* KA9)**	0.021±0.001^{b}	0.046±0.003^{cd}	0.063±0.003^{ab}	0.058±0.003^{a}	0.043±0.003^{ab}	0.046
**T3 (*P. fluorescens* PDS1)**	0.021±0.001^{b}	0.043±0.003^{c}	0.061±0.004^{c}	0.062±0.003^{a}	0.044±0.002^{ab}	0.046
**T4 (*B. subtilis* KA9 + RS*)***	0.022±0.001^{ab}	0.055±0.002^{bc}	0.072±0.002^{b}	0.057±0.003^{a}	0.039±0.002^{b}	0.049
**T5 (*P. fluorescens* + RS*)***	0.023±0.001^{ab}	0.062±0.003^{b}	0.082±0.004^{a}	0.044±0.003^{b}	0.049±0.002^{ab}	0.052
**T6(RS+ KA9+ PDS1)**	0.024±0.001^{ab}	0.081±0.002^{a}	0.063±0.003^{ab}	0.063±0.003^{a}	0.046±0.002^{ab}	0.048
**T7 (RS)**	0.022±0.001^{ab}	0.051±0.002^{cd}	0.063±0.003^{ab}	0.056±0.004^{a}	0.044±0.002^{ab}	0.047
**Mean value**	0.022	0.052	0.062	0.052	0.040	-
**C.D.**	0.002	0.008	0.009	0.009	0.006	-
**SE(m)**	0.001	0.003	0.003	0.003	0.002	-
**SE(d)**	0.001	0.004	0.004	0.004	0.003	-
**C.V.**	5.662	11.862	11.422	13.178	11.601	-
