# Supplementary material for: Unraveling Microbial Volatile Elicitors Using a Transparent Methodology for Induction of Systemic Resistance and Regulation of Antioxidant Genes at Expression Levels in Chili against Bacterial Wilt Disease
Source: Antioxidants (Basel). 2022 Feb 16;11(2):404. doi: 10.3390/antiox11020404 (PMC8869530; doi:10.3390/antiox11020404)
Supplement: Supplementary file 1 [file antioxidants-11-00404-s001.zip › supplementary Table S6.pdf]

Table 4.2.14. Supplementary Table S6 Effect of volatile compound produced by biocontrol agents on induction of Superoxide dismutase activities in chilli Roots against *R. solanacearum* in glasshouse condition

Treatments	Superoxide dismutase activity (ΔOD/min/mg protein)					
	**0 hapi**	**24hapi**	**48hapi**	**72hapi**	**96hapi**	**Mean value**
T1 (Control)	0.0027±0.02 b	0.0028±0.024e	0.0035±0.030 e	0.0031±0.027d	0.0027±0.026 d	0.0029
T2 (*B. subtilis*KA9)	0.0028±0.03 b	0.00534±0.029 d	0.0071±0.027c	0.0064±0.033a b	0.0043±0.034c	0.0051
T3 (*P. fluorescens* PDS1)	0.0027±0.03 b	0.0055±0.035d	0.0063±0.028 d	0.0061±0.031a b	0.0045±0.036 b	0.0050
T4 (*B. subtilis* KA9 + RS*)*	0.0033±0.03 a	0.0066±0.030c	0.0083±0.023 b	0.0068±0.030a	0.0052±0.040 a	0.0060
T5 (*P. fluorescens* +RS*)*	0.0028±0.03 b	0.0075±0.029a	0.0085±0.025 b	0.0055±0.034c	0.0045±0.033 b	0.0057
T6(RS+KA9+ PDS1)	0.0033±0.03 a	0.0072±0.031b	0.0091±0.026 a	0.0063±0.041a b	0.0047±0.031 b	0.0061
T7 (RS)	0.0029±0.03 b	0.0056±0.032d	0.0065±0.026c	0.0056±0.031c	0.0046±0.029 b	0.0050
Mean value	0.0029	0.0057	0.0070	0.0056	0.0043	-
