# Supplementary material for: Unraveling Microbial Volatile Elicitors Using a Transparent Methodology for Induction of Systemic Resistance and Regulation of Antioxidant Genes at Expression Levels in Chili against Bacterial Wilt Disease
Source: Antioxidants (Basel). 2022 Feb 16;11(2):404. doi: 10.3390/antiox11020404 (PMC8869530; doi:10.3390/antiox11020404)
Supplement: Supplementary file 1 [file antioxidants-11-00404-s001.zip › supplementary Table S7.pdf]

Table 4.2.15. Supplementary Table S7 Effect of volatile compound produce by biocontrol agents on induction of phenyl ammonium lyase (PAL) activities in chilli leaves against *R. solanacearum* under glasshouse condition.

**Treatment**	**PAL activity (µmole/ml)**					
	**0 hapi**	**24hapi**	**48hapi**	**72hapi**	**96hapi**	**Mean value**
T1 (Control)	37.666 ±1.24c	42.333 ±3.24c	49.333 ±2.56f	48.6667 ±2.11c	45.333 ±3.23d	44.52
T2 (*B. subtilis* KA9)	47.666±2.04b	65.000 ±3.20b	192.333 ±2.23e	136.333 ±2.34b	90.000 ±2.84c	106
T3 (*P. fluorescens* PDS1)	49.333±3.26b	66.333 ±2.22b	219.333 ±2.67d	145.333 ±2.28b	95.66667 ±2.18c	114.8
T4 (*B. subtilis* KA9 + RS*)*	52.0000 ±3.29b	77.333 ±2.23ab	245 .000±2.19c	191.933 ±2.34a	113.666 ±2.24b	135.6
T5 (*P. fluorescens* + RS*)*	62.000 ±2.24a	84.333 ±1.27a	265.000 ±1.64b	192.000 ±1.24a	123.333 ±2.84b	145.60
T6(RS+ KA9+ PDS1)	65.000 ±2.28a	88.333 ±3.24a	312.667 ±2.64a	207.000 ±3.12a	137 .000±2.37a	145.80
T7 (RS)	48.333 ±2.90b	63.666 ±2.25b	188.667 ±3.24e	134.333 ±2.21b	98.000 ±2.28c	106.8
Mean value	51.42	69.28	210.00	150.42	100.14	-
