# Supplementary material for: Unraveling Microbial Volatile Elicitors Using a Transparent Methodology for Induction of Systemic Resistance and Regulation of Antioxidant Genes at Expression Levels in Chili against Bacterial Wilt Disease
Source: Antioxidants (Basel). 2022 Feb 16;11(2):404. doi: 10.3390/antiox11020404 (PMC8869530; doi:10.3390/antiox11020404)
Supplement: Supplementary file 1 [file antioxidants-11-00404-s001.zip › supplementary Table S8.pdf]

Table 4.2.16. Supplementary Table S8 Effect of volatile compound produce by biocontrol agents on induction of phenyl ammonium lyase activities in chilli roots against *R. solanacearum* in glasshouse condition.

**Treatments**	**PAL activity (µmole/ml)**					
	**0 hapi**	**24hapi**	**48hapi**	**72hapi**	**96hapi**	**Mean value**
T1 (Control)	33.33±2.24a	35.00±1.89e	41.00±2.35e	38.00±2.00d	37.00±2.36f	36.80
T2 (*B. subtilis* KA9)	32.00±2.36a	61.00±2.56d	187.00±2.36d	133.00±3.08bc	83.00±2.36de	99.20
T3 (*P. fluorescens* PDS1)	32.67±2.55a	65.00±2.81cd	213.00±4.41c	141.67±2.73b	90.33±2.93cd	108.32
T4 (*B. subtilis* KA9 + RS*)*	34.67±1.874a	73.00±2.14bc	240.33±3.89b	185.33±2.84a	104.67±2.65bc	127.20
T5 (*P. fluorescens* + RS*)*	38.33±1.87a	80.33±2.17ab	254.67±3.16b	187.00±2.24a	115.67±3.85b	134.80
T6(RS+ KA9+ PDS1)	31.00±1.89a	88.00±2.00a	299.67±3.40a	199.33±3.42a	132.00±3.18a	149.80
T7 (RS)	36.00±1.89a	58.00±2.86d	184.67±2.65d	124.33±2.92c	72.33±2.35e	94.80
Mean value	33.71	65.71	202.57	143.85	90.42	-
