# Supplementary material for: Unraveling Microbial Volatile Elicitors Using a Transparent Methodology for Induction of Systemic Resistance and Regulation of Antioxidant Genes at Expression Levels in Chili against Bacterial Wilt Disease
Source: Antioxidants (Basel). 2022 Feb 16;11(2):404. doi: 10.3390/antiox11020404 (PMC8869530; doi:10.3390/antiox11020404)
Supplement: Supplementary file 1 [file antioxidants-11-00404-s001.zip › supplementary Table S9.pdf]

Table S9 4.3.2. Relative expression levels (log fold change) of defense-related genes by strains PDS1 and KA9 different treatments in chilli root tissue following rhizobacteria inoculation and pathogen challenge after 48 hours.

**Defense-related genes**	**Relative expression levels (log fold change) Minus ddCT**					
	**Mock**	**RS**	**KA9+ RS**	**PDS1 + RS**	**KA9+PDS1 + RS**	**CC**
PAL	0	2.54[a]	3.25[a]	3.09[a]	3.75[a]	4.78[b]
POD	0	2.26[b]	2.29[d]	2.6[b]	3.22[c]	3.5[f]
SOD	0	1.03[e]	2.46[c]	2.12[c]	3.57[b]	3.68[e]
WRKYa	0	0.64[h]	1.36[g]	0.82[f]	1.43[f]	2.66[i]
PAL1	0	1.51[d]	0.99[h]	1.58[d]	1.17[g]	5.04[a]
DEF1	0	1.62[c]	1.11[f]	0.48[g]	2.11[e]	4.03[c]
CAT2	0	-0.34[j]	2.6[b]	-0.34[k]	0.91[h]	3.75[d]
WRKY40	0	-0.05[i]	-0.23[j]	0.24[h]	0.91[h]	1.01[i]
HSFC1	0	1.08[f]	0.24[i]	-0.25[j]	0.9[h]	0.87[j]
LOX2	0	-0.98[k]	-0.93[k]	0.04[i]	0.84[i]	-0.63[k]
NPR1	0	0.93[g]	1.63[e]	1.22[e]	2.84[d]	3.13[g]
